# Longitudinal estimation of *Plasmodium falciparum* prevalence in relation to malaria prevention measures in six sub-Saharan African countries

**DOI:** 10.1186/s12936-017-2078-3

**Published:** 2017-10-27

**Authors:** Chris Drakeley, Salim Abdulla, Selidji Todagbe Agnandji, José Francisco Fernandes, Peter Kremsner, Bertrand Lell, Ludovic Mewono, Bache Emmanuel Bache, Michael Gabriel Mihayo, Omar Juma, Marcel Tanner, Marc Christian Tahita, Halidou Tinto, Salou Diallo, Palpouguini Lompo, Umberto D’Alessandro, Bernhards Ogutu, Lucas Otieno, Solomon Otieno, Walter Otieno, Janet Oyieko, Kwaku Poku Asante, Dominic Bon-Ereme Dery, George Adjei, Elisha Adeniji, Dorcas Atibilla, Seth Owusu-Agyei, Brian Greenwood, Samwel Gesase, John Lusingu, Coline Mahende, Robert Mongi, Method Segeja, Samuel Adjei, Tsiri Agbenyega, Alex Agyekum, Daniel Ansong, John Tanko Bawa, Harry Owusu Boateng, Léonard Dandalo, Veronica Escamilla, Irving Hoffman, Peter Maenje, Francis Martinson, Terrell Carter, Didier Leboulleux, David C. Kaslow, Effua Usuf, Jean-Yves Pirçon, Edith Roset Bahmanyar

**Affiliations:** 1 London School of Hygiene and Tropical Medicine, London, UK; 2 Ifakara Institute of Health, Bagamoyo Research and Training Centre, Bagamoyo District Hospital, P.O. Box 74, Bagamoyo, Tanzania; 3 Albert Schweitzer Hospital, Lambaréné, Gabon and Institute of Tropical Medicine, University of Tübingen, Tübingen, Germany; 4 Swiss Tropical and Public Health Institute, Basel, Switzerland; 5 University of Basel, Basel, Switzerland; 6 Institut de Recherche en Sciences de la Santé, Nanoro, Burkina Faso; 7 Medical Research Council Unit, The Gambia, Banjul, Gambia; 8 KEMRI-Walter Reed Project, Kombewa, Kenya; 9 Kintampo Health Research Center, Kintampo, Ghana; 10 National Institute for Medical Research, Korogwe, Tanzania; 11 Kwame Nkrumah University of Science and Technology, Agogo, Ghana; 12 University of North Carolina Project, Lilongwe, Malawi; 13 The PATH Malaria Vaccine Initiative, Washington, D.C. USA; 14 GSK Vaccines, Wavre, Belgium

**Keywords:** Epidemiology, Malaria, Transmission, Prevalence, *Plasmodium falciparum*, Anaemia

## Abstract

**Background:**

*Plasmodium falciparum* prevalence (*Pf*PR) is a widely used metric for assessing malaria transmission intensity. This study was carried out concurrently with the RTS,S/AS01 candidate malaria vaccine Phase III trial and estimated *Pf*PR over ≤ 4 standardized cross-sectional surveys.

**Methods:**

This epidemiology study (NCT01190202) was conducted in 8 sites from 6 countries (Burkina Faso, Gabon, Ghana, Kenya, Malawi, and Tanzania), between March 2011 and December 2013. Participants were enrolled in a 2:1:1 ratio according to age category: 6 months–4 years, 5–19 years, and ≥ 20 years, respectively, per year and per centre. All sites carried out surveys 1–3 while survey 4 was conducted only in 3 sites. Surveys were usually performed during the peak malaria parasite transmission season, in one home visit, when medical history and malaria risk factors/prevention measures were collected, and a blood sample taken for rapid diagnostic test, microscopy, and haemoglobin measurement. *Pf*PR was estimated by site and age category.

**Results:**

Overall, 6401 (survey 1), 6411 (survey 2), 6400 (survey 3), and 2399 (survey 4) individuals were included in the analyses. In the 6 months–4 years age group, the lowest prevalence (assessed using microscopy) was observed in 2 Tanzanian centres (4.6% for Korogwe and 9.95% for Bagamoyo) and Lambaréné, Gabon (6.0%), while the highest *PfPR* was recorded for Nanoro, Burkina Faso (52.5%). *PfPR* significantly decreased over the 3 years in Agogo (Ghana), Kombewa (Kenya), Lilongwe (Malawi), and Bagamoyo (Tanzania), and a trend for increased *Pf*PR was observed over the 4 surveys for Kintampo, Ghana. Over the 4 surveys, for all sites, *Pf*PR was predominantly higher in the 5–19 years group than in the other age categories. Occurrence of fever and anaemia was associated with high *P. falciparum* parasitaemia. Univariate analyses showed a significant association of anti-malarial treatment in 4 surveys (odds ratios [ORs]: 0.52, 0.52, 0.68, 0.41) and bed net use in 2 surveys (ORs: 0.63, 0.68, 1.03, 1.78) with lower risk of malaria infection.

**Conclusion:**

Local *Pf*PR differed substantially between sites and age groups. In children 6 months–4 years old, a significant decrease in prevalence over the 3 years was observed in 4 out of the 8 study sites.

*Trial registration* Clinical Trials.gov identifier: NCT01190202:NCT. GSK Study ID numbers: 114001

**Electronic supplementary material:**

The online version of this article (doi:10.1186/s12936-017-2078-3) contains supplementary material, which is available to authorized users.

## Background

The burden of malaria remains the highest in sub-Saharan Africa, with 90% of cases and 92% of malaria deaths occurring in this region alone in 2015 [1]. Reducing the burden relies, among other factors, on accurate estimations of malaria transmission intensity (MTI), which determines the average age of first exposure, the rate of development of immunity, and consequently the age pattern of clinical disease [2, 3]. An effective and readily available approach to evaluate MTI is by using the *Plasmodium falciparum* prevalence (*Pf*PR), defined as the prevalence of asexual blood stage infection in human hosts. *Pf*PR is a widely accepted and long-standing measurement of the level of malaria risk in a community, with proven biological, epidemiological and statistical properties and consistent with the historical hypo-, meso- and hyper-endemic malaria categorizations [4, 5]. *Pf*PR may vary seasonally, due to variable ecological conditions, and is influenced by the population immunity against malaria [6].

Between 2000 and 2015, malaria transmission in Africa, as assessed by *Pf*PR, has decreased to almost half and this was correlated with a 40% decrease in the incidence of clinical disease [7]. This was due to significant and consistent investment in campaigns to increase the coverage of malaria control interventions, among which the use of insecticide-treated nets (ITNs) was assessed as the most effective, followed by artemisinin-based combination therapy and indoor residual spraying (IRS) [7].

This study was designed to evaluate estimates of *Pf*PR using microscopy, over a period of 3 years in sites from several sub-Saharan African countries, concurrently with and in the same environs as (but independently from) a Phase III clinical trial of the GSK’s candidate malaria vaccine RTS,S/AS01 [8–11]. Associations between malaria infection and occurrence of fever and anaemia, use of malaria control measures, or presence of malaria risk factors were also assessed during the study. Standardized study procedures were used to facilitate comparisons between sites and to provide robust data for subsequent upload to global databases such as the Malaria Atlas Project [12]. Study methodologies closely followed those of the Malaria Indicator Survey [13].

## Methods

### Study design and survey population

**Fig. 1:**
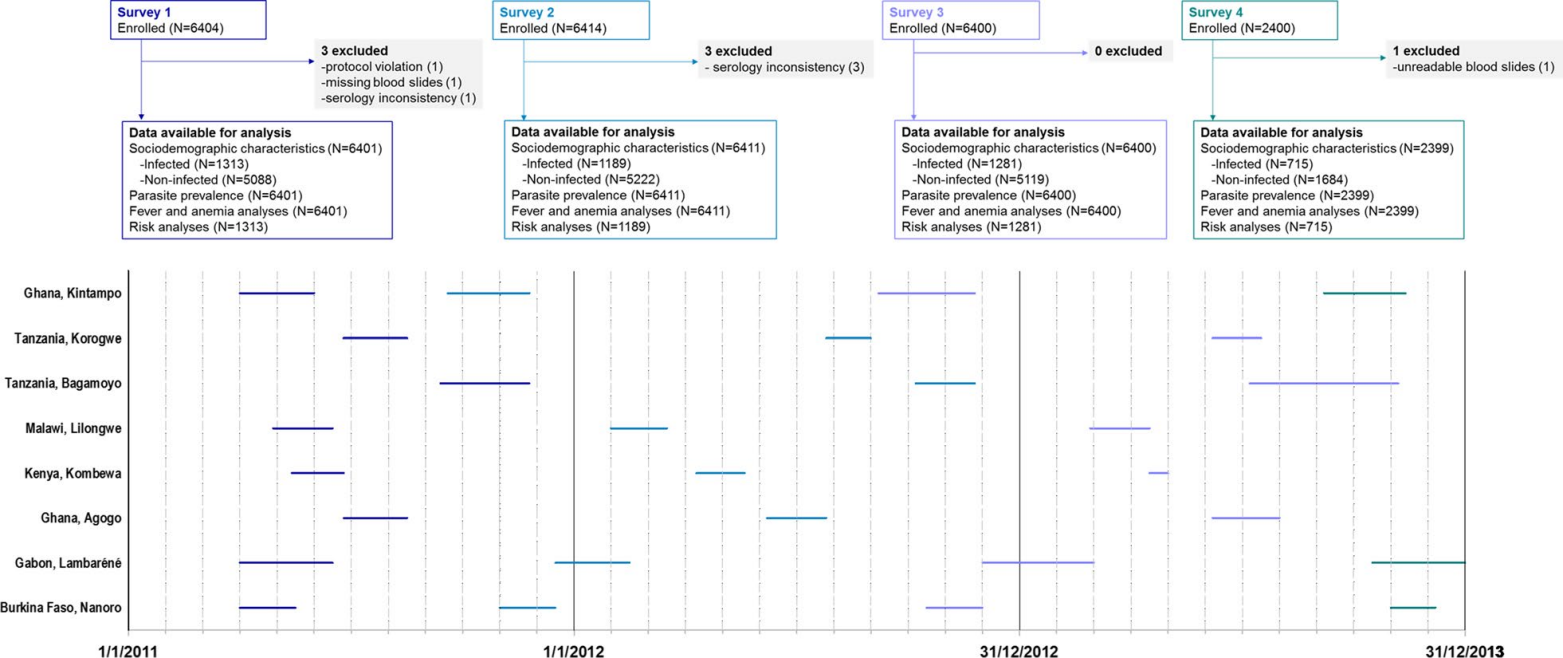
Flow diagram. *N* number of participants

This multicentre, epidemiology study was conducted between March 2011 and December 2013, and comprised up to 4 cross-sectional surveys. Among the 11 sites (distributed in 7 countries) which implemented the phase III trial of the candidate malaria vaccine RTS,S/AS01, 8 centres in Burkina Faso, Gabon, Ghana, Kenya, Malawi, and Tanzania agreed to participate and were included in this study. Five centres conducted 3 surveys and 3 centres (Nanoro in Burkina Faso, Lambaréné in Gabon, and Kintampo in Ghana) conducted 4 surveys, with the first 2 surveys carried out between March 2011 and January 2012 (Fig. 1). All surveys were conducted at the peak of malaria transmission (i.e. the end of rainy season) [8], except for the first surveys in the 3 centres that conducted 4 surveys.

Eligible participants were ≥ 6 months of age and not actively participating in either malaria vaccine or anti-malarial drug trials. Selection of study participants was based on either an existing demographic surveillance system, if available, or enumeration lists of all households in the study area. Children < 5 years (for the former) or households (for the latter) were randomly selected according to a computer-generated list. For each selected child ≥ 6 months to < 5 years old (6 months–4 years), another participant from the same household was considered for enrolment: either an individual 5–19 years old (5–19 years) or aged 20 years or more (≥ 20 years). To allow for a 10% non-response rate, 440 children 6 months–4 years were selected from the demographic surveillance system or, when unavailable, 660 houses were selected from enumeration lists, assuming 65–70% of houses will have children 6 months–4 years of age. To minimize sampling bias, 3 attempts were made to establish contact with a household before replacing it. If more than 1 child aged 6 months–4 years lived in the house, 1 was randomly selected.

### Ethical considerations

The trial protocol was approved by the ethical review board and national regulatory authorities at each study site and partner institutions. The survey was undertaken in accordance with the Good Clinical Practice Guidelines [14]. Depending on the age of the participants and local requirements, written informed consent and informed assent (where applicable) were obtained. Illiterate parents or legal representatives signed the consent form with a thumbprint, and a literate witness countersigned it. The study is registered at ClinicalTrials.gov (NCT01190202) and a summary of the protocol is available at http://www.gsk-clinicalstudyregister.com (ID: 114001).

### Data collection

Demographic information, medical history, socio-economic factors, and environmental and ecological factors were collected at each survey, during home visits. A blood sample was collected by finger prick for rapid diagnostic test, thin and thick blood film, haemoglobin measurement, and filter paper blood spot. Blood smears were examined by two independent microscopists, and discrepancies were settled by a third reader. Parasite density was estimated by examination of 100 high powered fields, either against measured blood volume [15] or against blood cell concentration [16] or by counting parasites against 200 white blood cells and assuming 8000 white blood cells/µl. Haemoglobin concentration was assessed using a spectrophotometer (Hemocue). Anaemia was managed according to national guidelines [17].

### Endpoints

Parasite density (parasites/μl) was defined as the geometric mean of 2 blood smear-reading values if the participant status was defined as positive. The parasite densities were grouped into the following predefined classes: low (< 2500 parasites/μl); medium (2500–9999 parasites/µl), high (10,000–19,999 parasites/μl), and very high (≥ 20,000 parasites/μl). Fever was defined as an axillary temperature ≥ 37.5 °C, or a history of fever reported in the 24 h prior to each survey. Anaemia was considered as non-severe for haemoglobin when between 7 g/dl and < 11–13 g/dl, depending on the age group (6 months–5, 5–11, 12–13, and > 13 years), gender and pregnancy status, and severe for a haemoglobin cut-off of < 7 g/dl, regardless of age and gender [18, 19]. Seroconversion rates, which were derived from age specific anti-malaria antibody prevalence [20], were also measured in the study and will be presented elsewhere.

### Statistical analysis

The sample size was computed for each centre separately and used to estimate point prevalence of infection among children in the 6 months–4 years group during one cross-sectional survey. The effective sample size was computed to obtain an estimation of the prevalence of infection with a relative standard error ≤ 0.25. To meet this criterion of precision, the effective sample size was estimated at 400 eligible participants per study centre. For participants in the 5–19 years or ≥ 20 years groups, a relative standard error ≤ 0.35 was considered acceptable, as the sample size of 200 eligible participants in both age categories per centre was assumed to be sufficient.

Analyses were carried out on data from all evaluable participants, i.e. those meeting all eligibility criteria, complying with protocol procedures and with available laboratory blood sample for *Pf*PR and serology data.

#### Demographics and survey data

Descriptive analyses were conducted for demographic and medical history characteristics, socio-economic factors and environmental and ecological factors. For each centre, unadjusted odds ratios (OR) with 95% confidence intervals (CIs) were computed for the association of malaria control measure or risk factor with the risk of *P. falciparum* infection.

#### Parasite prevalence and anaemia status

The analysis of *Pf*PR was conducted by year, site, age group and year of age for children younger than 5 years. Pooled prevalence across centres was done by age class using generalized estimating equations in order to take into account the correlation between participants from each site/country [21, 22], and heterogeneity among centers was tested using Cochran’s Q test based upon inverse variance weights.

Univariate analysis using logistic regression was conducted to determine the association between infection and anaemia, malaria control measures, or malaria risk factors. ORs (equal to the hazard ratio of the logistic regression) were estimated in the entire population for each centre separately and also adjusted with the centre as a random effect.

Statistical analyses were performed using SAS version 9.2.

## Results

### Study population

The number of participants enrolled and reasons for exclusion from analyses are presented in Fig. 1. Demographic and socio-economic characteristics of participants, as well as the use of malaria prevention measures are summarized in Table 1 for the 6 months–5 years age category. The number of enrolled participants and male to female ratio was similar between sites and over the 3 years. As study centres were classified as rural, semi-rural or urban, access to electricity, or source of drinking water varied substantially between centres (Table 1). Similarly, reported malaria treatment and use of preventive measures varied between centres, but were comparable for the same centre over the years (Table 1). The proportion of participants sleeping under a bed net the night prior to each survey varied greatly over the 3 years and across all age groups. For the 6 months–4 years age category, the lowest reported use of bed nets was reported in Nanoro (33.3%) and Kintampo (39.4%) for the first survey, but at survey 4 it increased up to 94.0 and 80.8% respectively (Table 1). Exposure to IRS in the 12 months prior to each survey was reported for a low proportion of participants; the highest proportion was documented in Agogo, and varied between 3.5% (survey 2) and 11.1% (survey 1) for households of children aged 6 months–4 years (Table 1).

**Table 1:** Demographic and socio-economic characteristics of survey participants in the 6 months–4 years age category, by study site

	Burkina Faso, Nanoro	Gabon, Lambaréné	Ghana, Agogo	Kenya, Kombewa	Malawi, Lilongwe	Tanzania, Bagamoyo	Tanzania, Korogwe	Ghana, Kintampo
Survey 1, N	423	398	397	400	400	392	389	401
Rural location, % (95% CI)	100 (99.1–100)	31.7 (27.1–36.5)	36.0 (31.3–41.0)	98.8 (97.1–99.6)	0.5 (0.1–1.8)	100 (99.1–100)	86.1 (82.3–89.4)	48.6 (43.6–53.6)
Closed water source, % (95% CI)	60.5 (55.7–65.2)	71.9 (67.2–76.2)	94.2 (91.4–96.3)	44.0 (39.1–49.0)	99.3 (97.8–99.8)	23.0 (18.9–27.4)	53.0 (47.9–58.0)	71.3 (66.6–75.7)
Electricity present, % (95% CI)	1.7 (0.7–3.4)	76.1 (71.6–80.2)	73.6 (68.9–77.8)	3.5 (1.9–5.8)	14.8 (11.4–18.6)	4.3 (2.5–6.9)	4.6 (2.8–7.2)	44.9 (39.9–49.9)
Malaria treatment in past 14 days, n (%)	2 (0.5)	7 (1.8)	58 (14.6)	86 (21.5)	54 (13.5)	12 (3.1)	28 (7.2)	60 (15.0)
Slept under bed net the night before, n (%)	141 (33.3)	292 (73.4)	149 (37.5)	324 (81.0)	227 (56.8)	371 (94.6)	275 (70.7)	158 (39.4)
No use of repellent in past 7 days, n (%)	416 (98.3)	187 (47.0)	229 (57.7)	390 (97.5)	390 (97.5)	388 (99.0)	367 (94.3)	296 (73.8)
IRS in past 12 months, n (%)	0 (0.0)	1 (0.3)	31 (7.8)	4 (1.0)	16 (4.0)	0 (0.0)	0 (0.0)	7 (1.7)
Insecticide spray use in past 7 days, n (%)	3 (0.7)	16 (4.0)	44 (11.1)	3 (0.8)	1 (0.3)	1 (0.3)	4 (1.0)	29 (7.2)
Survey 2, N	402	398	400	400	399	398	399	401
Rural location, % (95% CI)	100 (99.1–100)	22.6 (18.6–27.0)	0.0 (0.0–0.9)	96.3 (93.9–97.9)	0.0 (0.0–0.9)	84.9 (81.0–88.3)	84.2 (80.3–87.6)	67.1 (62.2–71.7)
Closed water source, % (95% CI)	73.4 (68.8–77.6)	76.4 (71.9–80.5)	97.5 (95.5–98.8)	43.8 (38.8–48.8)	98.2 (96.4–99.3)	32.4 (27.8–37.3)	63.4 (58.5–68.1)	57.9 (52.9–62.7)
Electricity present, % (95% CI)	1.1 (0.3–2.5)	84.9 (81.0–88.3)	65.8 (60.9–70.4)	8.8 (6.2–12.0)	19.0 (15.3–23.3)	1.5 (0.6–3.3)	5.0 (3.1–7.6)	41.6 (36.8–46.6)
Malaria treatment in past 14 days, n (%)	4 (1.0)	11 (2.8)	48 (12.0)	75 (18.8)	23 (5.8)	14 (3.5)	21 (5.3)	88 (21.9)
Slept under bed net the night before, n (%)	293 (72.9)	296 (74.4)	285 (71.3)	367 (91.8)	251 (62.9)	358 (89.9)	363 (91.0)	206 (51.4)
No use of repellent in past 7 days, n (%)	402 (100)	202 (50.8)	316 (79.0)	389 (97.3)	314 (78.7)	390 (98.0)	391 (98.0)	322 (80.3)
IRS in past 12 months, n (%)	0 (0.0)	0 (0.0)	1 (0.3)	8 (2.0)	5 (1.3)	1 (0.3)	0 (0.0)	0 (0.0)
Insecticide spray use in past 7 days, n (%)	0 (0.0)	10 (2.5)	14 (3.5)	4 (1.0)	16 (4.0)	2 (0.5)	1 (0.3)	13 (3.2)
Survey 3, N	395	399	399	401	400	399	400	400
Rural location, % (95% CI)	100 (99.1–100)	35.8 (31.1–40.8)	13.8 (10.6–17.6)	95.3 (92.7–97.1)	0.0 (0.0–0.9)	100 (99.1–100)	89.0 (85.5–91.9)	82.0 (77.9–85.6)
Closed water source, % (95% CI)	75.9 (71.4–80.1)	62.4 (57.5–67.2)	90.5 (87.2–93.2)	30.7 (26.2–35.4)	98.5 (96.8–99.4)	39.1 (34.3–44.1)	44.5 (39.6–49.5)	56.0 (51.0–60.9)
Electricity present, % (95% CI)	2.3 (1.0–4.3)	68.9 (64.1–73.4)	74.9 (70.4–79.1)	8.7 (6.2–11.9)	21.0 (17.1–25.3)	1.3 (0.4–2.9)	5.8 (3.7–8.5)	31.3 (26.7–36.0)
Malaria treatment in past 14 days, n (%)	32 (8.1)	2 (0.5)	52 (13.0)	78 (19.5)	26 (6.5)	10 (2.5)	36 (9.0)	73 (18.3)
Slept under bed net the night before, n (%)	352 (89.1)	250 (62.7)	242 (60.7)	362 (90.3)	330 (82.5)	362 (90.7)	339 (84.8)	348 (87.0)
No use of repellent in past 7 days, n (%)	387 (98.0)	165 (41.4)	304 (76.2)	387 (96.5)	383 (95.8)	395 (99.0)	388 (97.0)	369 (92.3)
IRS in past 12 months, n (%)	0 (0.0)	0 (0.0)	0 (0.0)	4 (1.0)	0 (0.0)	1 (0.3)	0 (0.0)	2 (0.5)
Insecticide spray use in past 7 days, n (%)	0 (0.0)	4 (1.0)	21 (5.3)	6 (1.5)	8 (2.0)	3 (0.8)	3 (0.8)	3 (0.8)
Survey 4, N	399	399	–	–	–	–	–	395
Rural location, % (95% CI)	100 (99.1–100)	35.1 (30.4–40.0)	–	–	–	–	–	82.3 (78.1–85.9)
Closed water source, % (95% CI)	87.5 (83.8–90.6)	74.2 (69.6–78.4)	–	–	–	–	–	71.4 (66.7–75.8)
Electricity present, % (95% CI)	2.0 (0.9–3.9)	76.7 (72.2–80.8)	–	–	–	–	–	24.8 (20.6–29.4)
Malaria treatment in past 14 days, n (%)	29 (7.3)	1 (0.3)	–	–	–	–	–	54 (13.7)
Slept under bed net the night before, n (%)	375 (94.0)	212 (53.1)	–	–	–	–	–	319 (80.8)
No use of repellent in past 7 days, n (%)	393 (98.5)	164 (41.1)	–	–	–	–	–	348 (88.1)
IRS in past 12 months, n (%)	1 (0.3)	0 (0.0)	–	–	–	–	–	2 (0.5)
Insecticide spray use in past 7 days, n (%)	3 (0.8)	10 (2.5)	–	–	–	–	–	14 (3.5)

Closed water source: piped water, tube well, dug well, protected well. No use of repellent in past 7 days: no use of mosquito coil, insecticide spray, commercial or traditional repellent. Percentages were computed without considering the missing values

*M* months of age, *Y* years of age, *N* number of participants included in the analyses, *n (%)* number/percentage of participants in a given category, *CI* confidence interval, *IRS* indoor residual spray, *–* centres not included in the fourth survey

Additional file 1: Tables S1 and S2 summarize the data for the 5–19 and ≥ 20 years age groups.

### *Plasmodium falciparum* prevalence (*PfPR*)

Within each age group, *Pf*PR varied substantially between centres. No consistent patterns of change over the 3 years could be identified across age groups (Table 2).

**Table 2:** *Plasmodium falciparum* prevalence by site, age group and year

	Burkina Faso, Nanoro	Gabon, Lambaréné	Ghana, Agogo	Kenya, Kombewa	Malawi, Lilongwe	Tanzania, Bagamoyo	Tanzania, Korogwe	Ghana, Kintampo	Pooled (GEE model)
Survey 1									
6 months–4 years	52.5 (47.6–57.3)	6.0 (3.9–8.8)	23.2 (19.1–27.6)	43.8 (38.8–48.8)	11.5 (8.5–15.0)	10.0 (7.2–13.4)	4.6 (2.8–7.2)	25.9 (21.7–30.5)	22.2 (12.7–35.8)
5–19 years	59.1 (51.6–66.4)	10.3 (6.6–15.2)	19.7 (14.5–25.9)	53.4 (46.3–60.4)	19.0 (13.8–25.1)	16.0 (11.1–21.9)	6.6 (3.7–10.9)	41.2 (34.4–48.3)	28.2 (17.0–42.8)
≥ 20 years	14.9 (10.2–20.7)	5.0 (2.3–9.2)	6.0 (3.1–10.3)	17.4 (12.3–23.4)	9.5 (5.8–14.4)	3.7 (1.6–7.1)	2.5 (0.8–5.7)	15.9 (11.1–21.8)	9.3 (6.1–14.0)
Survey 2									
6 months–4 years	67.7 (62.9–72.2)	7.3 (4.9–10.3)	19.5 (15.7–23.7)	22.8 (18.7–27.2)	6.3 (4.1–9.1)	2.0 (0.9–3.9)	1.0 (0.3–2.6)	16.7 (13.2–20.7)	17.9 (7.7–36.2)
5–19 years	81.8 (75.7–86.9)	15.9 (11.2–21.7)	28.4 (22.2–35.1)	42.0 (35.1–49.2)	14.4 (9.9–20.1)	6.9 (3.9–11.1)	2.5 (0.8–5.7)	42.0 (35.1–49.2)	29.2 (15.5–48.2)
≥ 20 years	32.0 (25.6–39.0)	7.2 (4.0–11.8)	6.5 (3.5–10.9)	9.0 (5.4–13.9)	4.5 (2.1–8.4)	2.1 (0.6–5.2)	1.0 (0.1–3.6)	11.1 (7.1–16.3)	9.2 (4.5–17.8)
Survey 3									
6 months–4 years	66.3 (61.4–71.0)	9.0 (6.4–12.3)	15.0 (11.7–18.9)	27.4 (23.1–32.1)	3.5 (1.9–5.8)	1.3 (0.4–2.9)	2.5 (1.2–4.6)	27.0 (22.7–31.6)	19.0 (8.6–37.0)
5–19 years	79.6 (73.5–84.9)	17.3 (12.4–23.3)	19.8 (14.5–26.0)	54.2 (47.1–61.3)	9.0 (5.4–13.9)	1.0 (0.1–3.4)	4.8 (2.2–8.9)	51.5 (44.4–58.5)	29.7 (14.8–50.6)
≥ 20 years	42.7 (35.7–49.9)	8.0 (4.7–12.7)	1.5 (0.3–4.3)	18.2 (13.1–24.3)	7.5 (4.3–12.1)	1.6 (0.3–4.5)	2.4 (0.8–5.4)	15.5 (10.7–21.3)	12.2 (5.6–24.3)
Survey 4									
6 months–4 years	53.4 (48.4–58.4)	6.8 (4.5–9.7)	–	–	–	–	–	26.6 (22.3–31.2)	28.9 (12.4–53.8)
5–19 years	70.7 (63.8–76.8)	12.6 (8.3–18.1)	–	–	–	–	–	53.6 (46.6–60.6)	45.6 (21.7–71.8)
≥ 20 years	25.5 (19.6–32.1)	3.0 (1.1–6.4)	–	–	–	–	–	17.7 (12.6–23.7)	15.4 (7.5–29.1)

Pooled (GEE model), estimated proportion using generalized estimating equations with centre as random effect

*M* months of age, *Y* years of age, *–* centres not included in the fourth survey

In the 6 months–4 years age group, the estimated pooled *Pf*PR varied in time between 17.9% and 28.9%. In the 5–19 years age group, pooled *Pf*PRs across the 3 years were in the 28.2–45.6% range, while in participants ≥ 20 years of age, pooled *Pf*PR varied over the 3 years between 9.2% and 15.4% (Table 2). Heterogeneity across centres for *Pf*PR was significant in each age category (Cochran’s Q test p < 0.0001).

**Fig. 2:**
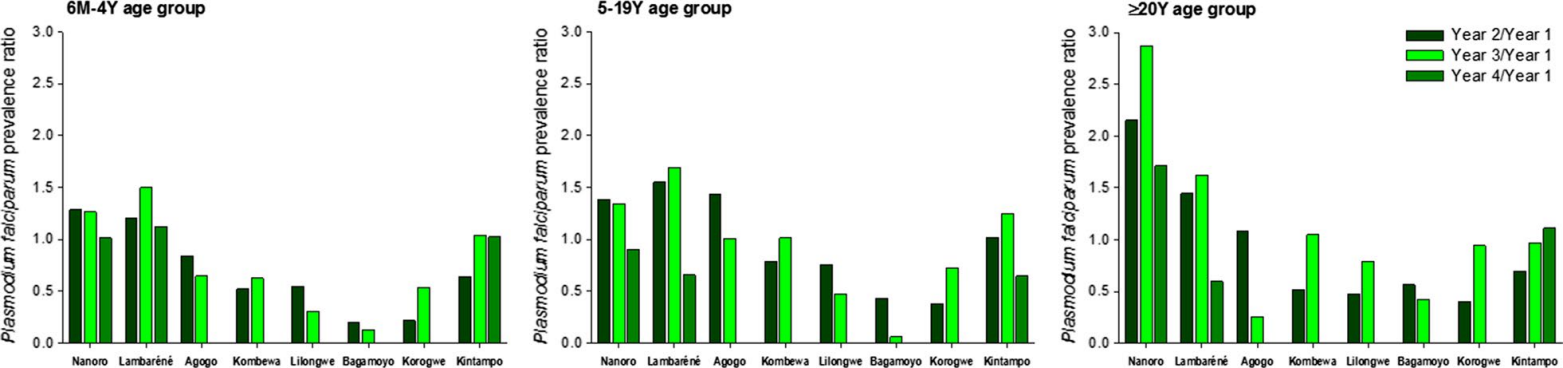
Decrease of *Plasmodium falciparum* prevalence over the study period, by site and age group. *M* month, *Y* year

**Fig. 3:**
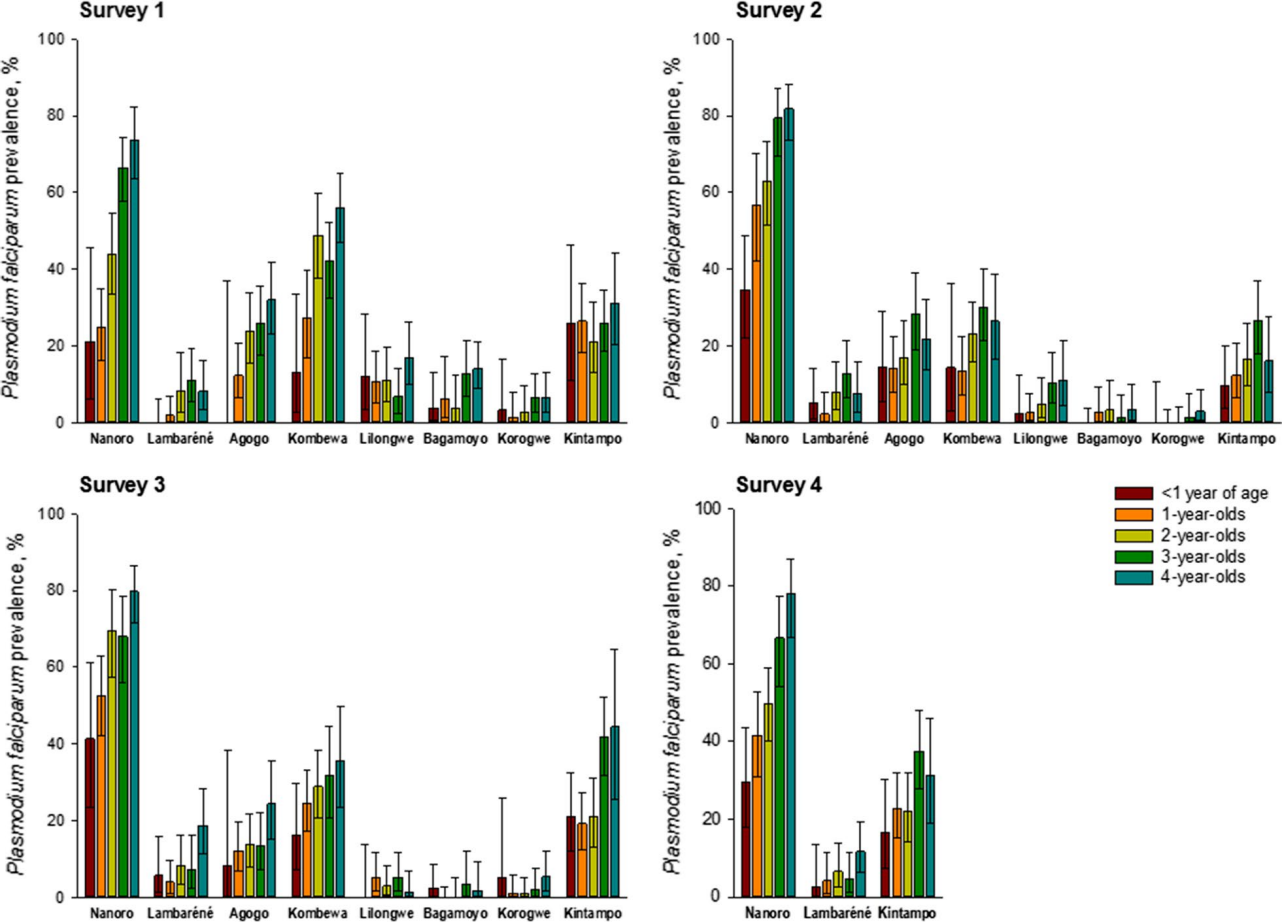
*Plasmodium falciparum* prevalence by age, in the 6 months–4 years category, by site and study year. *M* month, *Y* year

In the 6 months–4 years age category, at survey 1, the lowest prevalence was observed in the 2 Tanzanian centres (4.6% for Korogwe and 10.0% for Bagamoyo) and Lambaréné, Gabon (6.0%). The highest *Pf*PR was recorded in Nanoro (52.5%). In this age group and at the time of survey 3, the infection prevalence significantly decreased in Agogo (Ghana), Kombewa (Kenya), Lilongwe (Malawi), and Bagamoyo (Tanzania), as shown by non-overlapping 95% CI. A trend for decreased *Pf*PR was observed over the last 3 surveys for Nanoro, while in Kintampo, *Pf*PR tended to increase over the last 3 surveys (Table 2, Fig. 2). Within the 6 months–4 years category, *Pf*PR increased with age (Fig. 3).

Across all centres and years, *PfPR*s were higher in the 5–19 years age group compared to the 6 months–4 years group. The lowest *Pf*PRs in individuals aged 5–19 years were recorded at the 2 Tanzanian sites, for which a significant decrease was documented from survey 1 to survey 2. The highest values were observed for Nanoro, with a *Pf*PR of 59.1% at survey 1 and 70.7% at survey 4, but showing a decrease if only the last 3 surveys were considered, with a peak at survey 2 (*Pf*PR = 81.8%). *Pf*PR was also high in Kombewa, varying from 42.0% (survey 2) to 54.2% (survey 3) over the 3 surveys conducted. A trend for increasing *Pf*PR was observed for Kintampo, while *Pf*PR decreased in Lilongwe over the study period (Table 2).

*Plasmodium falciparum* prevalence was significantly lower and varied less by site in adults aged ≥ 20 years compared with the other age categories. In Nanoro, a significant increase in prevalence was observed from the time of survey 1 (14.9%) to surveys 2 (32.0%) and 3 (42.7%), but *PfPR* decreased again to 25.5% at the time the fourth survey was conducted. High prevalence in adults was also observed in Kintampo, and was relatively stable over the years (between 11.1 and 17.7%), while a trend for decreasing *Pf*PR was observed for Bagamoyo (Table 2).

Similar percentages of *P. falciparum*-infected individuals were also observed by rapid diagnostic test (Additional file 2: Table S1).

Infection by *Plasmodium malariae*, *Plasmodium vivax* and *Plasmodium ovale* was rarely observed in all sites across all surveys, in less than 5% of the participants (Additional file 2: Table S2).

### Occurrence of fever and anaemia

**Fig. 4:**
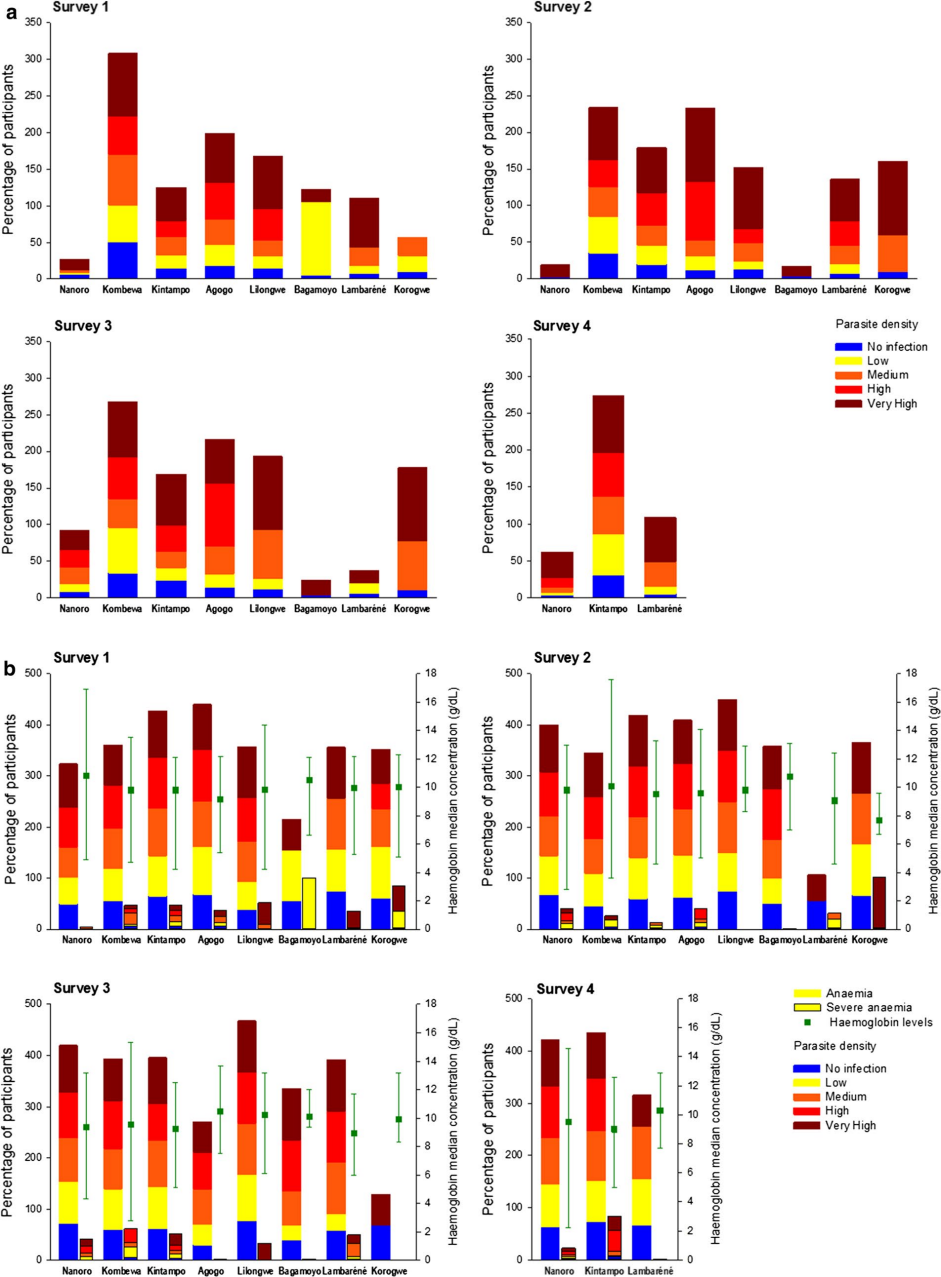
Prevalence of fever (**a**), anaemia and severe anaemia and haemoglobin concentration (**b**) for the 6 months–4 years age category, by parasite-density category and site. *M* month, *Y* year. Prevalence was calculated as the percentage of children with reported fever/anaemia/severe anaemia in each parasite-density category. The sites are ordered according to increasing values of *Plasmodium falciparum* prevalence in the first survey

The percentage of participants of all ages reporting fever in the 24 h prior to each survey was significantly higher in *P. falciparum*-infected than in non-infected individuals: 26.0% (95% CI 23.7–28.5) versus 13.1% (95% CI 12.2–14.0) for survey 1, 17.7% (95% CI 15.6–20.0) versus 12.3% (95% CI 11.4–13.2) for survey 2, 23.8% (95% CI 21.5–26.2) versus 12.1% (95% CI 11.2–13.0) for survey 3, and 21.5% (95% CI 18.6–24.7) versus 9.6% (95% CI 8.3–11.1) for survey 4. The same trend was observed in each age group (Fig. 4a; Additional file 1: Figure S1). The proportion of participants with reported fever varied greatly between centres for all age groups.

In the 6 months–4 years age group, occurrence of fever (measured or reported with the last 24 h) tended to be higher in children with higher parasite density, although this differed by site (Fig. 4a). Prevalence of fever was lower in Nanoro when compared with other sites with high *Pf*PR, for each of the 4 surveys. Occurrence of fever in the other age groups also increased with increasing parasite density, although this trend varied greatly across centres (Additional file 1: Figure S1).

Anaemia occurred more frequently in *P. falciparum*-infected compared to non-infected individuals (Fig. 4b; Additional file 1: Figure S2); prevalence of anaemia did not appear to be related to *Pf*PR. Severe anaemia [19] was reported in 5.4–9.6, 0.8–2.4 and 0.0–1.4% of infected participants in each of the 3 age groups, respectively.

*Plasmodium falciparum* infection was a significant risk factor for anaemia (p < 0.001), with estimated centre adjusted ORs of 1.89 (95% CI 1.47–2.35) for survey 1, 1.64 (95% CI 1.40–1.90) for survey 2, 1.61 (95% CI 1.19–2.19) for survey 3, and 1.76 (95% CI 1.40–2.19) for survey 4. ORs varied by site and study survey. For survey 3, infection was not associated with anaemia in Bagamoyo, Tanzania (OR = 0.30; 95% CI 0.08–1.17). For all other sites and surveys, OR values ranged between 1.21 (95% CI 0.89–1.65) and 4.08 (95% CI 0.88–18.98) (Additional file 3: Table S1).

### Malaria control measures and risk factors for malaria infection

Reported use of preventive measures varied substantially across study centres (Table 1; Additional file 3: Table S2). Having slept under a bed net the night before the survey visit was significantly associated with lower odds of infection in the first 2 surveys, with OR = 0.63 (95% CI 0.56–0.71) for survey 1 and 0.68 (95% CI 0.60–0.78) for survey 2, but not for the last 2 surveys (OR = 1.03 [95% CI 0.89–1.20] for survey 3 and 17.8 [95% CI 1.44–2.20] for survey 4) (Table 3). Risk of malaria infection was lower in participants who reported having received an anti-malarial treatment in the previous 14 days; OR values ranged between 0.41 and 0.68 across the 3 years. Household IRS in the 12 months prior to the survey was only evaluated in centres where exposure to IRS was reported, but overall, it was associated with decreased odds of infection by *P. falciparum* (Table 3). Reported personal use of insecticide sprays was also associated with lower odds of infection over the years, with ORs varying between 0.39 and 0.57 (Table 3).

**Table 3:** Odds ratios analysis of *P. falciparum* infection in relation to reported usage of antimalarial treatment and prevention measures

	Odds ratio (95% confidence interval)			
	Survey 1	Survey 2	Survey 3	Survey 4
Malaria treatment in past 14 days (yes vs. no)	0.52 (0.0–0.68)	0.52 (0.38–0.69)	0.68 (0.52–0.89)	0.41 (0.24–0.70)
Slept under bed net night before (yes vs. no)	0.63 (0.56–0.71)	0.68 (0.60–0.78)	1.03 (0.89–1.20)	1.78 (1.44–2.20)
Use of at least one of the following over 7 days: mosquito coils—insecticide spray—commercial or traditional repellents	0.44 (0.36–0.54)	0.60 (0.49–0.74)	0.54 (0.44–0.67)	0.25 (0.19–0.33)
IRS in past 12 months^a^ (yes vs. no)	0.58 (0.33–0.99)	0.31 (0.07–1.31)	0.80 (0.18–3.65)	1.18 (0.22–6.45)
Insecticide spray use in past 7 days (yes vs. no)	0.39 (0.24–0.64)	0.43 (0.23–0.80)	0.57 (0.31–1.04)	0.49 (0.17–0.90)

*IRS* indoor residual spray

^a^To spray interior walls

## Discussion

This study showed that, among regions where the RTS,S/AS01 Phase III clinical trial was implemented, *Pf*PR differed greatly by age and site; nevertheless, a higher malaria prevalence in the 5–19 years age group was observed consistently in all sites. Burkina Faso was the country with the highest *Pf*PRs over the 3 years, while the lowest prevalence was consistently observed in the 2 Tanzanian sites and Lambaréné in Gabon. In children aged 6 months–4 years, the age group for which the highest morbidity and mortality due to malaria is observed [23], a significant decrease in *Pf*PR was observed over time at 3 sites: Kombewa (Kenya), Lilongwe (Malawi) and Bagamayo (Tanzania). The use of anti-malarial treatment was associated with reduced odds of *P. falciparum* infection for all sites.

The higher *Pf*PR observed in Burkina Faso is not surprising and is in line with previous reports [24]. In Nouna, Burkina Faso, malaria prevalence in children under 5 was 57.7% [24], similar to that observed in Nanoro in our study. The results also confirm a lower *Pf*PR in the younger 6 months–5 years age group compared with children over 5 years of age reported in Nouna [24]. A decline of *Pf*PR over time was observed when the surveys at the same 3 time points were considered, in line with reports of a consistently decreased prevalence between 2009 and 2011, compared to 2000 [25]. A high prevalence was also observed in Kombewa, Kenya, for which data for the last 2 surveys seems to be in line with previous reports in a similar timeframe and geographical setting for children 0–5 years [26], although larger values were observed in our study for the other age groups. Among the sites in Ghana, lower *Pf*PR values in Agogo than in Kintampo were consistently documented in our study. This difference is not surprising, as marked heterogeneity of *P. falciparum* infection was previously reported [27].

The lower *P. falciparum* prevalence noted in the Tanzanian centres is in agreement with recent estimations of *Pf*PR by rapid diagnostic test in a community survey [28], and reports of a decline in malaria incidence in this country, following scale up of malaria control interventions [29]. Lilongwe, Malawi was the only other site where a consistent decrease over time was observed in, both for the 6 months–5 years and the 5–19 years age groups. The estimated *Pf*PRs compared fairly well with the *P. falciparum* infection prevalence detected microscopically from a study conducted in southern Malawi from 2012 to 2014, although larger values were reported in the same study when real-time polymerase chain reaction (PCR) was used for parasite detection [30]. In Lambaréné, the estimated *Pf*PR was lower in the 6 months–5 years age group than in the 5–19 years one, which is consistent with previous observation of an increase in age of the malaria high-risk population in Gabon between 2008 and 2011 [31].

Ultimately, the *Pf*PRs’ heterogeneity across the 8 study sites represents well the variability of transmission intensity in sub-Saharan Africa [32, 33], and confirms that RTS,S/AS01 vaccine efficacy was evaluated at varying levels of MTI [34].

Differences across centres were also observed in relation to the use of anti-malarials and control measures, but they are also likely to be associated with other extrinsic factors such as social, behavioural, economic and local policies. Previous anti-malarial treatment (in all surveys) and use of bed nets (in ≥ 2 surveys) was associated with a lower risk of infection for the majority of the sites. During the study, the proportion of participants having taken malaria treatment varied widely in centres with high malaria prevalence; the lowest recorded use of malaria treatment was in Nanoro, Burkina Faso (0.5–2.5% of participants) and the highest in Kintampo, Ghana (up to 21.9% of participants). Of note, to our knowledge, Seasonal Malaria Chemoprevention measures as recommended by the World Health Organization had not been implemented at the time the study ended in any of the study sites [35]. This finding may be explained by a number of highly interrelated extrinsic environmental, social, and economic factors such as diagnosis confirmation and local treatment practices, care seeking behaviour, access to health facilities and availability of local anti-malarials [36]. The discrepancy between infection prevalence and infrequent use of anti-malarials at some sites may be related to the immunity afforded by chronic infections resulting in asymptomatic parasite carriage, which have been widely described in *P. falciparum* endemic areas, including Africa [37].

Bed net use (on the night before the survey) was high in all sites throughout the 4 years and was associated with reduced risk of infection. Although recent data confirm the effectiveness of ITNs [7], reports in the literature from the period covered by our study did not always evidence an association between bed net use and lower odds of infection at all sites. For instance, while a national distribution campaign in Burkina Faso led to an increase in the use of ITNs, the more frequent use of bed nets was not associated with a decline in parasitaemia in children under 5 years of age [38], as was the case in The Gambia [27]. In Malawi, use of ITN following a high coverage of malaria interventions was associated with protection against infection, but *P. falciparum* prevalence remained high, especially in school-aged children [30].

Increased use of ITNs and IRS has already been associated with a reduction in malaria burden through a population effect, and the fact that the results of these measures have varied across regions [39–47] and by age group [30] is mainly due to issues in their implementation, although differences in the dominant malaria vector species could also play a role [48]. Of note, in this study, information on using malaria medication or control measures was based on questions such as sleeping under a bed net the night before the survey, taking anti-malarial medication during the past 14 days, or exposure to IRS during the past 12 months, which may induce recall biases or generalize usage of bed net based on the report for 1 night. Moreover, malaria control interventions may be targeted to areas with higher parasite prevalence and this, together with variations in coverage within an area, may confound any observations.

As expected, both anaemia and fever were associated with *P. falciparum*-infection in this study. OR analyses confirmed *P. falciparum* infection to be a significant risk factor for anaemia, with ORs ranging from 1.21 to 4.08 across all sites and surveys (notwithstanding the negative association found in Bagamoyo at survey 3), consistent with results from other studies [49–51].

Overall, fever was associated with high parasite densities (≥ 10,000 parasites/μl). There was a trend towards a higher association between fever and parasite density in the youngest age group, but results varied widely across centres. The lower fever prevalence observed in Nanoro, the site with the highest overall *Pf*PR for all surveys and age groups, seems to be in line with previous findings in Burkina Faso, with 85–90% of parasite-positive individuals showing no fever [25]. However, in sites with consistently low *Pf*PR for the 6 months–4 years age group (Lambaréné, Lilongwe, Korogwe, Kintampo), fever prevalence was also substantial for low to medium parasite densities (< 10,000 parasites/μl). These results show that a lower parasite density was associated with fever at sites where *Pf*PR (and therefore MTI) was lower, as children in regions with moderate-to-high levels of MTI may develop partial immunity to malaria more easily and at an early age, manifested through a decrease in symptoms [37, 52]. Of note, a larger prevalence of fever, regardless of recorded parasitaemia, was observed in Kintampo compared with the other study sites; moreover, in the 6 months–4 years age group, fever was reported for 31.0–50.2% of non-infected children. However, this observation is in agreement with reports of a high incidence of non-malaria fever in children born between 2008 and 2011 in the area [53].

A number of key factors contributed to the quality and reliability of results presented in this paper. Standard methodologies were used across centres to ensure comparability of results and to make certain that sampling was representative across catchment areas which participated in the Phase III trial. This allowed for a surplus in the number of randomly selected households in order to take into account non-response and to minimize sampling bias. However, the sampling followed a non-probability method, as the targeted population represented a convenience sample, and therefore, generalization of the results should be made with caution.

The study has several limitations. Four surveys were conducted only in 3 study sites, as the study ended in December 2013, along with the Phase III trial, and at that time, 5 centres had not conducted the fourth survey. For some centres, the first survey was carried out outside the peak transmission season. *P. falciparum* prevalence was assessed microscopically, a method which underestimates the true level of infection when compared with molecular (PCR) detection [28, 54]. The cut-offs used to define anaemia and severe anaemia were based on the haemoglobin levels at sea level as recommended by the World Health Organization, but were not adjusted for each site, and this could have impacted the estimation of anaemia prevalence.

Malaria control intervention may have a different effect and/or impact on malaria morbidity at different levels of MTI. Therefore, the assessment of MTI in parallel to efficacy trials for new malaria control interventions is essential for the generalization of trial results to other settings and to guide their implementation [55–58]. When administered as a 4-dose schedule at months 0, 1, 2 and 20, the vaccine efficacy of RTS,S/AS01 against clinical malaria estimated in the Phase III trial was 39.0% (95% CI 34.3–43.3), over a median follow-up period of 48 months since first vaccination of children 5–17 months old [59], up to the year 2014. However, vaccine efficacy varied greatly among study sites, although there was no evidence for a statistically significant interaction with MTI [10]. Of note, ordering the sites by increasing *Pf*PR for the 4 study years provided a similar ranking as when using incidence of clinical malaria, measured in control infants 6–12 weeks of age at enrollment in the Phase III study during 12 months of follow-up [10, 11], especially for the 6 months–4 years and 5–19 years age categories. For all 4 years in our study, the site with the highest *Pf*PR was Nanoro, for which the highest malaria incidence was observed in the Phase III trial, while the lowest values were documented for Korogwe in both studies. The order of the other sites also compared fairly between *Pf*PR and clinical incidence of malaria, suggesting that the control group could serve as a surrogate for relative malaria parasite transmission intensity. The data in our study contribute to an improved interpretation of site variations in the Phase III vaccine efficacy study of RTS,S/AS01.

## Additional files

**Additional file 1.** Data for 5–19 and ≥ 20 years age categories.

**Additional file 2.** Malaria rapid diagnostic test results and prevalence of infections with other parasites.

**Additional file 3.** Odds ratios analysis of *P. falciparum* infection in relation to reported usage of anti-malarial treatment and prevention.

## Abbreviations

CI: confidence interval
IRS: indoor residual spray
ITN: insecticide-treated net
M: month
MTI: malaria transmission intensity
OR: odds ratio
*Pf*PR: *Plasmodium falciparum* prevalence
Y: year

## References

[1] WHO. Malaria fact sheet. Geneva: World Health Organization. http://www.who.int/mediacentre/factsheets/fs094/en/. Accessed 22 Mar 2017.

[2] Snow RW, Marsh K (2002). The consequences of reducing transmission of *Plasmodium falciparum* in Africa. Adv Parasitol.

[3] Snow RW, Omumbo JA, Lowe B, Molyneux CS, Obiero JO, Palmer A (1997). Relation between severe malaria morbidity in children and level of *Plasmodium falciparum* transmission in Africa. Lancet.

[4] Hay SI, Smith DL, Snow RW (2008). Measuring malaria endemicity from intense to interrupted transmission. Lancet Infect Dis..

[5] Smith DL, Guerra CA, Snow RW, Hay SI (2007). Standardizing estimates of the *Plasmodium falciparum* parasite rate. Malar J..

[6] O’Meara WP, Collins WE, McKenzie FE (2007). Parasite prevalence: a static measure of dynamic infections. Am J Trop Med Hyg.

[7] Bhatt S, Weiss DJ, Cameron E, Bisanzio D, Mappin B, Dalrymple U (2015). The effect of malaria control on *Plasmodium falciparum* in Africa between 2000 and 2015. Nature.

[8] The RTS,S Clinical Trials Partnership (2011). First results of phase 3 trial of RTS,S/AS01 malaria vaccine in African children. N Engl J Med.

[9] The RTS,S Clinical Trials Partnership (2012). A phase 3 trial of RTS,S/AS01 malaria vaccine in African infants. N Engl J Med.

[10] The RTS,S Clinical Trials Partnership (2014). Efficacy and safety of the RTS,S/AS01 malaria vaccine during 18 months after vaccination: a phase 3 randomized, controlled trial in children and young infants at 11 African sites. PLoS Med..

[11] The RTS,S Clinical Trials Partnership (2015). Efficacy and safety of RTS,S/AS01 malaria vaccine with or without a booster dose in infants and children in Africa: final results of a phase 3, individually randomised, controlled trial. Lancet.

[12] Malaria Atlas Project. http://www.map.ox.ac.uk/. Accessed 2 Mar 2017.

[13] Malaria Indicators Surveys. http://malariasurveys.org/index.cfm. Accessed 2 Mar 2017.

[14] International Conference on Harmonization of Technical Requirements for Registration of Pharmaceuticals for Human Use (ICH): Guidance for industry: E6 good clinical practice: consolidated guidance. 1996. 38–42: 50–8. https://www.ich.org/fileadmin/Public_Web_Site/ICH_Products/Guidelines/Efficacy/E6/E6_R1_Guideline.pdf. Accessed 22 Mar 2017.

[15] Planche T, Krishna S, Kombila M, Engel K, Faucher JF, Ngou-Milama E (2001). Comparison of methods for the rapid laboratory assessment of children with malaria. Am J Trop Med Hyg.

[16] Greenwood BM, Armstrong JR (1991). Comparison of two simple methods for determining malaria parasite density. Trans R Soc Trop Med Hyg.

[17] Ministry of Health, Republic of Ghana. Standard treatment guidelines. 6th ed. 2010. http://apps.who.int/medicinedocs/documents/s18015en/s18015en.pdf. Accessed 22 Mar 2017.

[18] United Nations Children’s Fund, United Nations University, World Health Organization. Iron deficiency anaemia assessment, prevention, and control—a guide for programme managers (WHO/NHD/01.3). 2001. http://www.who.int/nutrition/publications/en/ida_assessment_prevention_control.pdf. Accessed 4 April 2017.

[19] Stoltzfus RJ, Dreyfuss ML. Guidelines for the use of iron supplements to prevent and treat iron deficiency anemia. Washington DC, ILSI PRESS; 1993. http://www.who.int/nutrition/publications/micronutrients/guidelines_for_Iron_supplementation.pdf. Accessed 2 Mar 2017.

[20] Corran P, Coleman P, Riley E, Drakeley C (2007). Serology: a robust indicator of malaria transmission intensity?. Trends Parasitol..

[21] Freeman MF, Tukey JW (1950). Transformations related to the angular and the square root. Ann Math Stat..

[22] Miller JJ (1978). The inverse of the Freeman-Tukey double arcsine transformation. Am Stat.

[23] WHO. Malaria in children under five. Geneva: World Health Organization. http://www.who.int/malaria/areas/high_risk_groups/children/en/. Accessed 22 Mar 2017.

[24] Diallo A, Sie A, Sirima S, Sylla K, Ndiaye M, Bountogo M (2017). An epidemiological study to assess *Plasmodium falciparum* parasite prevalence and malaria control measures in Burkina Faso and Senegal. Malar J..

[25] Geiger C, Agustar HK, Compaore G, Coulibaly B, Sie A, Becher H (2013). Declining malaria parasite prevalence and trends of asymptomatic parasitaemia in a seasonal transmission setting in North-Western Burkina Faso between 2000 and 2009–2012. Malar J..

[26] Idris ZM, Chan CW, Kongere J, Gitaka J, Logedi J, Omar A (2016). High and heterogeneous prevalence of asymptomatic and sub-microscopic malaria infections on Islands in Lake Victoria, Kenya. Sci Rep..

[27] Mwesigwa J, Okebe J, Affara M, Di Tanna GL, Nwakanma D, Janha O (2015). On-going malaria transmission in The Gambia despite high coverage of control interventions: a nationwide cross-sectional survey. Malar J..

[28] Mwingira F, Genton B, Kabanywanyi A-NM, Felger I (2014). Comparison of detection methods to estimate asexual *Plasmodium falciparum* parasite prevalence and gametocyte carriage in a community survey in Tanzania. Malar J..

[29] Tanzania Commission for AIDS (TACAIDS) ZAC. Tanzania HIV/AIDS and Malaria Indicator Survey 2011–2012. Dar es Salaam, Tanzania: TACAIDS, ZAC, NBS, OCGS, and ICF International; 2013. http://tacaids.go.tz/tacaids/index.php/component/phocadownload/category/12-front-end-documents?download=44:thmis-final-report&lang=en. Accessed 23 Mar 2017.

[30] Buchwald AG, Coalson JE, Cohee LM, Walldorf JA, Chimbiya N, Bauleni A (2017). Insecticide-treated net effectiveness at preventing *Plasmodium falciparum* infection varies by age and season. Malar J..

[31] Mawili-Mboumba DP, Bouyou Akotet MK, Kendjo E, Nzamba J, Medang MO, Mbina JR (2013). Increase in malaria prevalence and age of at risk population in different areas of Gabon. Malar J..

[32] Kelly-Hope LA, McKenzie FE (2009). The multiplicity of malaria transmission: a review of entomological inoculation rate measurements and methods across sub-Saharan Africa. Malar J..

[33] Shaukat AM, Breman JG, McKenzie FE (2010). Using the entomological inoculation rate to assess the impact of vector control on malaria parasite transmission and elimination. Malar J..

[34] Leach A, Vekemans J, Lievens M, Ofori-Anyinam O, Cahill C, Owusu-Agyei S (2011). Design of a phase III multicenter trial to evaluate the efficacy of the RTS, S/AS01 malaria vaccine in children across diverse transmission settings in Africa. Malar J..

[35] Olumese P. Seasonal Malaria Chemoprevention: WHO policy and perspectives. ACCESS SMC Meeting. Transforming the malaria landscape in the Sahel: seasonal malaria chemoprevention. 9 June 2016, London, UK. http://www.malariaconsortium.org/media-downloads/825/SMC%20-%20WHO%20policy%20and%20perspectives. Accessed 4 Apr 2017.

[36] Yakasai AM, Hamza M, Dalhat MM, Bello M, Gadanya MA, Yaqub ZM (2015). Adherence to artemisinin-based combination therapy for the treatment of uncomplicated malaria: a systematic review and meta-analysis. J Trop Med..

[37] Lindblade KA, Steinhardt L, Samuels A, Kachur SP, Slutsker L (2013). The silent threat: asymptomatic parasitemia and malaria transmission. Expert Rev Anti Infect Ther..

[38] Louis VR, Schoeps A, Tiendrebeogo J, Beiersmann C, Ye M, Damiba MR (2015). An insecticide-treated bed-net campaign and childhood malaria in Burkina Faso. Bull World Health Organ.

[39] Asante KP, Zandoh C, Dery DB, Brown C, Adjei G, Antwi-Dadzie Y (2011). Malaria epidemiology in the Ahafo area of Ghana. Malar J..

[40] Binka FN, Kubaje A, Adjuik M, Williams LA, Lengeler C, Maude GH (1996). Impact of permethrin impregnated bednets on child mortality in Kassena-Nankana district, Ghana: a randomized controlled trial. Trop Med Int Health..

[41] Fegan GW, Noor AM, Akhwale WS, Cousens S, Snow RW (2007). Effect of expanded insecticide-treated bednet coverage on child survival in rural Kenya: a longitudinal study. Lancet.

[42] Lindblade KA, Eisele TP, Gimnig JE, Alaii JA, Odhiambo F, ter Kuile FO (2004). Sustainability of reductions in malaria transmission and infant mortality in western Kenya with use of insecticide-treated bednets: 4 to 6 years of follow-up. JAMA.

[43] Lindblade KA, Gimnig JE, Kamau L, Hawley WA, Odhiambo F, Olang G (2006). Impact of sustained use of insecticide-treated bednets on malaria vector species distribution and culicine mosquitoes. J Med Entomol.

[44] Namountougou M, Diabate A, Etang J, Bass C, Sawadogo SP, Gnankinie O (2013). First report of the L1014S kdr mutation in wild populations of *Anopheles gambiae* M and S molecular forms in Burkina Faso (West Africa). Acta Trop.

[45] Namountougou M, Simard F, Baldet T, Diabate A, Ouedraogo JB, Martin T (2012). Multiple insecticide resistance in *Anopheles gambiae* s.l. populations from Burkina Faso, West Africa. PLoS ONE.

[46] Otten M, Aregawi M, Were W, Karema C, Medin A, Bekele W (2009). Initial evidence of reduction of malaria cases and deaths in Rwanda and Ethiopia due to rapid scale-up of malaria prevention and treatment. Malar J..

[47] Zhou G, Afrane YA, Vardo-Zalik AM, Atieli H, Zhong D, Wamae P (2011). Changing patterns of malaria epidemiology between 2002 and 2010 in Western Kenya: the fall and rise of malaria. PLoS ONE.

[48] Lengeler C. Insecticide-treated bed nets and curtains for preventing malaria. Cochrane Database Syst Rev. 2004;(2):CD000363. doi:10.1002/14651858.CD000363.pub2 10.1002/14651858.CD000363.pub2 15106149

[49] Clarke SE, Brooker S, Njagi JK, Njau E, Estambale B, Muchiri E (2004). Malaria morbidity among school children living in two areas of contrasting transmission in western Kenya. Am J Trop Med Hyg.

[50] Marsh K, Snow RW (1999). Malaria transmission and morbidity. Parassitologia..

[51] Saute F, Aponte J, Almeda J, Ascaso C, Abellana R, Vaz N (2003). Malaria in southern Mozambique: malariometric indicators and malaria case definition in Manhica district. Trans R Soc Trop Med Hyg.

[52] Filipe JA, Riley EM, Drakeley CJ, Sutherland CJ, Ghani AC (2007). Determination of the processes driving the acquisition of immunity to malaria using a mathematical transmission model. PLoS Comput Biol.

[53] Asante KP, Owusu-Agyei S, Cairns M, Boamah E, Manu G, Twumasi M (2016). Non-malaria fevers in a high malaria endemic area of Ghana. BMC Infect Dis.

[54] Parr JB, Belson C, Patel JC, Hoffman IF, Kamthunzi P, Martinson F (2016). Estimation of *Plasmodium falciparum* transmission intensity in Lilongwe, Malawi, by microscopy, rapid diagnostic testing, and nucleic acid detection. Am J Trop Med Hyg.

[55] WHO. Malaria vector control and personal protection: Report of a WHO study group. Geneva: World Health Organization; 2006. p. 62. WHO Technical Report series No. 936. 16623084

[56] Greenwood BM, Bojang K, Whitty CJ, Targett GA (2005). Malaria. Lancet.

[57] Shiff C (2002). Integrated approach to malaria control. Clin Microbiol Rev.

[58] Snow RW, Marsh K, le Sueur D (1996). The need for maps of transmission intensity to guide malaria control in Africa. Parasitol Today..

[59] Vandoolaeghe P, Schuerman L (2016). The RTS, S/AS01 malaria vaccine in children 5 to 17 months of age at first vaccination. Expert Rev Vaccines..

