# Supplementary material for: Longitudinal estimation of Plasmodium falciparum prevalence in relation to malaria prevention measures in six sub-Saharan African countries
Source: Malar J. 2017 Oct 27;16:433. doi: 10.1186/s12936-017-2078-3 (PMC5658967; doi:10.1186/s12936-017-2078-3)
Supplement: Supplementary file 1 — Additional file 1. Data for 5–19 and ≥ 20 years age categories. [file 12936_2017_2078_MOESM1_ESM.doc]

# Additional file 1. Data for the 5–19Y and 20Y age groups

**Table S1.** Demographic and socio-economic characteristics of survey participants by site, for the 5–19Y and ≥20Y age groups

|  |  |  | Burkina Faso, Nanoro | |  | Gabon, Lambaréné |  | Ghana,  Agogo |  | Kenya, Kombewa |  | Malawi, Lilongwe |  | Tanzania, Bagamoyo |  | Tanzania, Korogwe |  | Ghana,  Kintampo |  |
| --- | --- | --- | --- | --- | --- | --- | --- | --- | --- | --- | --- | --- | --- | --- | --- | --- | --- | --- | --- |
| Survey 1 | |  |  | |  |  |  |  |  |  |  |  |  |  |  |  |  |  |  |
| 5–19Y | Rural location, % (95% CI) |  | 100 (98.0–100) | |  | 31.5 (25.3–38.2) |  | 34.5 (28.0–41.5) |  | 98.5 (95.8–99.7) |  | 1.0 (0.1–3.6) |  | 100 (98.1–100) |  | 88.2 (83.0–92.2) |  | 64.7 (57.7–71.3) |  |
|  | Closed water source, % (95% CI)^a^ |  | 61.3 (53.8–68.5) | |  | 72.8 (66.3–78.6) |  | 95.1 (91.1–97.6) |  | 40.8 (34.0–47.8) |  | 99.5 (97.2–100) |  | 13.4 (8.9–19) |  | 55.0 (48.0–61.8) |  | 67.6 (60.8–74) |  |
|  | Electricity present, % (95% CI) |  | 2.2 (0.6–5.6) | |  | 76.1 (69.7–81.6) |  | 74.4 (67.8–80.2) |  | 2.4 (0.8–5.6) |  | 19.0 (13.8–25.1) |  | 2.6 (0.8–5.9) |  | 5.2 (2.6–9.1) |  | 37.7 (31.1–44.8) |  |
| ≥20Y | Rural location, % (95% CI) |  | 100 (98.1–100) | |  | 32.0 (25.3–39.4) |  | 36.5 (29.8–43.6) |  | 99.0 (96.4–99.9) |  | 0.0 (0.0–1.8) |  | 100 (98.3–100) |  | 85.6 (79.9–90.1) |  | 63.1 (55.9–69.9) |  |
|  | Closed water source, % (95% CI)^a^ |  | 62.1 (54.8–68.9) | |  | 70.2 (62.9–76.7) |  | 92.0 (87.3–95.4) |  | 46.9 (39.8–54.2) |  | 99.0 (96.4–99.9) |  | 32.9 (26.7–39.5) |  | 54.2 (47.1–61.3) |  | 69.7 (62.8–76.1) |  |
|  | Electricity present, % (95% CI) |  | 1.5 (0.3–4.4) | |  | 77.9 (71.1–83.7) |  | 73.0 (66.3–79.0) |  | 4.6 (2.1–8.5) |  | 12.0 (7.8–17.3) |  | 5.5 (2.9–9.4) |  | 4.5 (2.1–8.3) |  | 39.5 (32.6–46.7) |  |
| Survey 2 | |  |  | |  |  |  |  |  |  |  |  |  |  |  |  |  |  |  |
| 5–19Y | Rural location, % (95% CI) |  | 100 (98.2–100) | |  | 17.9 (12.9–23.8) |  | 0.0 (0.0–1.8) |  | 98.0 (95.0–99.5) |  | 0.0 (0.0–1.8) |  | 94.5 (90.6–97.1) |  | 84.1 (78.3–88.8) |  | 51.5 (44.3–58.6) |  |
|  | Closed water source, % (95% CI)^a^ |  | 72.7 (66.0–78.8) | |  | 77.8 (71.5–83.2) |  | 97.0 (93.6–98.9) |  | 41.0 (34.1–48.2) |  | 97.5 (94.3–99.2) |  | 27.9 (22.0–34.3) |  | 59.7 (52.6–66.5) |  | 59.0 (51.8–65.9) |  |
|  | Electricity present, % (95% CI) |  | 1.5 (0.3–4.4) | |  | 86.5 (81.0–90.8) |  | 66.7 (59.7–73.1) |  | 4.5 (2.1–8.4) |  | 15.4 (10.7–21.2) |  | 1.8 (0.5–4.6) |  | 5.5 (2.8–9.6) |  | 41.0 (34.1–48.2) |  |
| ≥20Y | Rural location, % (95% CI) |  | 100 (98.2–100) | |  | 27.2 (21.1–34.0) |  | 0.0 (0.0–1.8) |  | 94.5 (90.4–97.2) |  | 0.0 (0.0–1.8) |  | 76.3 (69.7–82.1) |  | 84.5 (78.7–89.2) |  | 59.3 (52.1–66.2) |  |
|  | Closed water source, % (95% CI)^a^ |  | 74.0 (67.3–79.9) | |  | 75.4 (68.7–81.3) |  | 97.5 (94.2–99.2) |  | 46.5 (39.4–53.7) |  | 99.0 (96.4–99.9) |  | 36.1 (29.3–43.3) |  | 66.5 (59.5–73.0) |  | 51.8 (44.6–58.9) |  |
|  | Electricity present, % (95% CI) |  | 0.5 (0.0–2.8) | |  | 83.6 (77.6–88.5) |  | 68.3 (61.4–74.7) |  | 13.0 (8.7–18.5) |  | 23.0 (17.4–29.5) |  | 1.0 (0.1–3.7) |  | 4.5 (2.1–8.4) |  | 41.7 (34.8–48.9) |  |
| Survey 3 | |  |  |  | | | | | | | | | | | | | | | |
| 5–19Y | Rural location, % (95% CI) |  | 100 (98.2–100) | |  | 32.2 (25.8–39.1) |  | 13.9 (9.4–19.4) |  | 95.5 (91.7–97.9) |  | 0.0 (0.0–1.8) |  | 100 (98.2–100) |  | 90.9 (85.8–94.6) |  | 84.5 (78.8–89.1) |  |
|  | Closed water source, % (95% CI)^a^ |  | 75.7 (69.3–81.4) | |  | 68.8 (61.9–75.1) |  | 90.1 (85.1–93.8) |  | 31.8 (25.5–38.8) |  | 98.5 (95.7–99.7) |  | 35.6 (29.1–42.5) |  | 42.2 (35.1–49.7) |  | 57.3 (50.2–64.1) |  |
|  | Electricity present, % (95% CI) |  | 3.4 (1.4–6.9) | |  | 75.7 (69.2–81.5) |  | 72.8 (66.1–78.8) |  | 11.9 (7.8–17.2) |  | 19.0 (13.8–25.1) |  | 1.0 (0.1–3.4) |  | 5.9 (3.0–10.3) |  | 25.7 (19.9–32.3) |  |
| ≥20Y | Rural location, % (95% CI) |  | 100 (98.2–100) | |  | 39.2 (32.4–46.3) |  | 13.6 (9.1–19.1) |  | 94.9 (90.9–97.6) |  | 0.0 (0.0–1.8) |  | 100 (98.1–100) |  | 87.3 (82.1–91.5) |  | 82.5 (76.4–87.5) |  |
|  | Closed water source, % (95% CI)^a^ |  | 77.9 (71.5–83.5) | |  | 56.3 (49.1–63.3) |  | 89.9 (84.9–93.8) |  | 28.8 (22.6–35.6) |  | 98.5 (95.7–99.7) |  | 42.5 (35.4–49.8) |  | 46.5 (39.6–53.4) |  | 59.3 (52.0–66.3) |  |
|  | Electricity present, % (95% CI) |  | 2.0 (0.6–5.1) | |  | 62.3 (55.2–69.1) |  | 75.4 (68.8–81.2) |  | 5.6 (2.8–9.7) |  | 23.0 (17.4–29.5) |  | 1.6 (0.3–4.5) |  | 5.6 (2.9–9.6) |  | 30.4 (24–37.4) |  |
| Survey 4 | |  |  | |  |  |  | - |  | - |  | - |  | - |  | - |  |  |  |
| 5-9Y | Rural location, % (95% CI) |  | 100 (98.2–100) | |  | 35.4 (28.7–42.4) |  | - |  | - |  | - |  | - |  | - |  | 83.6 (77.8–88.3) |  |
|  | Closed water source, % (95% CI)^a^ |  | 87.1 (81.6–91.4) | |  | 72.7 (66.0–78.8) |  | - |  | - |  | - |  | - |  | - |  | 68.6 (61.8–74.9) |  |
|  | Electricity present, % (95% CI) |  | 3.0 (1.1–6.4) | |  | 75.3 (68.6–81.1) |  | - |  | - |  | - |  | - |  | - |  | 21.3 (15.9–27.5) |  |
| ≥20Y | Rural location, % (95% CI) |  | 100 (98.2–100) | |  | 34.7 (28.1–41.7) |  | - |  | - |  | - |  | - |  | - |  | 86.9 (81.4–91.2) |  |
|  | Closed water source, % (95% CI)^a^ |  | 87.5 (82.1–91.7) | |  | 75.7 (69.2–81.5) |  | - |  | - |  | - |  | - |  | - |  | 68.2 (61.2–74.6) |  |
|  | Electricity present, % (95% CI) |  | 1.5 (0.3–4.3) | |  | 78.2 (71.9–83.7) |  | - |  | - |  | - |  | - |  | - |  | 28.3 (22.1–35.1) |  |

Y, years of age; %, percentage of participants in a given category; CI, confidence interval; -, centres not included in the fourth survey.

Note: ^a^ Piped water, tube well, dug well, protected well.

**Table S2**. Number and percentage of participants using malaria treatment and control measures by site, for the 5–19Y and ≥20Y age groups

|  |  | Burkina Faso, Nanoro |  | Gabon, Lambaréné |  | Ghana,  Agogo |  | Kenya, Kombewa |  | Malawi, Lilongwe |  | Tanzania, Bagamoyo |  | Tanzania, Korogwe |  | Ghana, Kintampo |  |
| --- | --- | --- | --- | --- | --- | --- | --- | --- | --- | --- | --- | --- | --- | --- | --- | --- | --- |
| 5–19Y |  |  |  |  |  |  |  |  |  |  |  |  |  |  |  |  |  |
| Survey 1, N |  | 181 |  | 214 |  | 203 |  | 206 |  | 200 |  | 194 |  | 211 |  | 204 |  |
| Malaria treatment in past 14 days^a^ |  | 1 (0.6) |  | 7 (3.3) |  | 12 (5.9) |  | 27 (13.1) |  | 20 (10.0) |  | 4 (2.1) |  | 19 (9.0) |  | 12 (5.9) |  |
| Slept under bed net the night before |  | 47 (26.0) |  | 125 (58.4) |  | 38 (18.7) |  | 144 (69.9) |  | 81 (40.5) |  | 177 (91.2) |  | 113 (53.6) |  | 54 (26.5) |  |
| No use of repellent in past 7 days^b^ |  | 179 (98.9) |  | 99 (46.3) |  | 117 (57.6) |  | 201 (97.6) |  | 194 (97.0) |  | 192 (99.0) |  | 199 (94.3) |  | 155 (76.0) |  |
| Use of IRS in past 12 months^c^ |  | 0 (0.0) |  | 0 (0.0) |  | 17 (8.4) |  | 2 (1.0) |  | 4 (2.0) |  | 1 (0.5) |  | 0 (0.0) |  | 0 (0.0) |  |
| Insecticide spray use in past 7 days |  | 2 (1.1) |  | 6 (2.8) |  | 21 (10.3) |  | 2 (1.0) |  | 1 (0.5) |  | 0 (0.0) |  | 2 (0.9) |  | 11 (5.4) |  |
| Survey 2, N |  | 198 |  | 207 |  | 201 |  | 200 |  | 201 |  | 219 |  | 201 |  | 200 |  |
| Malaria treatment in past 14 days^a^ |  | 1 (0.5) |  | 14 (6.8) |  | 11 (5.5) |  | 28 (14.0) |  | 8 (4.0) |  | 4 (1.8) |  | 10 (5.0) |  | 21 (10.5) |  |
| Slept under bed net night before^a^ |  | 130 (65.7) |  | 138 (66.7) |  | 100 (49.8) |  | 166 (83.0) |  | 91 (45.3) |  | 192 (87.7) |  | 171 (85.1) |  | 49 (24.5) |  |
| No use of repellent in past 7 days^b^ |  | 198 (100) |  | 102 (49.3) |  | 156 (77.6) |  | 195 (97.5) |  | 159 (79.1) |  | 218 (99.5) |  | 199 (99.0) |  | 154 (77.0) |  |
| Use of IRS in past 12 months^c^ |  | 0 (0.0) |  | 0 (0.0) |  | 1 (0.5) |  | 5 (2.5) |  | 2 (1.0) |  | 0 (0.0) |  | 0 (0.0) |  | 0 (0.0) |  |
| Insecticide spray use in past 7 days |  | 0 (0.0) |  | 5 (2.4) |  | 6 (3.0) |  | 2 (1.0) |  | 7 (3.5) |  | 1 (0.5) |  | 1 (0.5) |  | 10 (5.0) |  |
| Survey 3, N |  | 206 |  | 202 |  | 202 |  | 201 |  | 200 |  | 208 |  | 187 |  | 206 |  |
| Malaria treatment in past 14 days^a^ |  | 3 (1.5) |  | 0 (0.0) |  | 12 (5.9) |  | 26 (12.9) |  | 7 (3.5) |  | 3 (1.4) |  | 11 (5.9) |  | 16 (7.8) |  |
| Slept under bed net night before^a^ |  | 179 (86.9) |  | 110 (54.5) |  | 77 (38.1) |  | 160 (79.6) |  | 140 (70.0) |  | 180 (86.5) |  | 135 (72.2) |  | 147 (71.4) |  |
| No use of repellent in past 7 days^b^ |  | 202 (98.1) |  | 86 (42.6) |  | 152 (75.2) |  | 192 (95.5) |  | 189 (94.5) |  | 208 (100) |  | 181 (96.8) |  | 191 (92.7) |  |
| Use of IRS in past 12 months^c^ |  | 0 (0.0) |  | 0 (0.0) |  | 0 (0.0) |  | 2 (1.0) |  | 0 (0.0) |  | 0 (0.0) |  | 0 (0.0) |  | 1 (0.5) |  |
| Insecticide spray use in past 7 days |  | 0 (0.0) |  | 2 (1.0) |  | 7 (3.5) |  | 4 (2.0) |  | 3 (1.5) |  | 0 (0.0) |  | 2 (1.1) |  | 2 (1.0) |  |
| Survey 4, N |  | 201 |  | 189 |  |  |  |  |  |  |  |  |  |  |  | 207 |  |
| Malaria treatment in past 14 days^a^ |  | 5 (2.5) |  | 1 (0.5) |  | - |  | - |  | - |  | - |  | - |  | 6 (2.9) |  |
| Slept under bed net night before^a^ |  | 185 (92.0) |  | 94 (47.5) |  | - |  | - |  | - |  | - |  | - |  | 155 (74.9) |  |
| No use of repellent in past 7 days^b^ |  | 197 (98.0) |  | 78 (39.4) |  | - |  | - |  | - |  | - |  | - |  | 185 (89.4) |  |
| Use of IRS in past 12 months^c^ |  | 1 (0.5) |  | 0 (0.0) |  | - |  | - |  | - |  | - |  | - |  | 0 (0.0) |  |
| Insecticide spray use in past 7 days |  | 2 (1.0) |  | 5 (2.5) |  | - |  | - |  | - |  | - |  | - |  | 3 (1.4) |  |
| ≥20Y |  |  |  |  |  |  |  |  |  |  |  |  |  |  |  |  |  |
| Survey 1, N |  | 195 |  | 182 |  | 200 |  | 196 |  | 200 |  | 219 |  | 201 |  | 195 |  |
| Malaria treatment in past 14 days^a^ |  | 1 (0.5) |  | 2 (1.1) |  | 20 (10.0) |  | 26 (13.3) |  | 9 (4.5) |  | 9 (4.1) |  | 12 (6.0) |  | 24 (12.3) |  |
| Slept under bed net night before |  | 60 (30.8) |  | 133 (73.1) |  | 75 (37.5) |  | 171 (87.2) |  | 112 (56.0) |  | 204 (93.2) |  | 135 (67.2) |  | 68 (34.9) |  |
| No use of repellent in past 7 days^b^ |  | 193 (99.0) |  | 89 (48.9) |  | 112 (56.0) |  | 191 (97.4) |  | 195 (97.5) |  | 217 (99.1) |  | 191 (95.0) |  | 158 (81.0) |  |
| Use of IRS in past 12 months^c^ |  | 0 (0.0) |  | 1 (0.5) |  | 15 (7.5) |  | 2 (1.0) |  | 10 (5.0) |  | 1 (0.5) |  | 0 (0.0) |  | 3 (1.5) |  |
| Insecticide spray use in past 7 days |  | 1 (0.5) |  | 10 (5.5) |  | 20 (10.0) |  | 1 (0.5) |  | 0 (0.0) |  | 1 (0.5) |  | 2 (1.0) |  | 11 (5.6) |  |
| Survey 2, N |  | 200 |  | 195 |  | 199 |  | 200 |  | 200 |  | 194 |  | 200 |  | 199 |  |
| Malaria treatment in past 14 days^a^ |  | 1 (0.5) |  | 5 (2.6) |  | 14 (7.0) |  | 19 (9.5) |  | 2 (1.0) |  | 7 (3.6) |  | 13 (6.5) |  | 27 (13.6) |  |
| Slept under bed net night before^a^ |  | 137 (68.5) |  | 140 (71.8) |  | 139 (69.8) |  | 189 (94.5) |  | 129 (64.5) |  | 179 (92.3) |  | 176 (88) |  | 62 (31.2) |  |
| No use of repellent in past 7 days^b^ |  | 200 (100) |  | 100 (51.3) |  | 163 (81.9) |  | 194 (97.0) |  | 158 (79.0) |  | 189 (97.4) |  | 194 (97.0) |  | 152 (76.4) |  |
| Use of IRS in past 12 months^c^ |  | 0 (0.0) |  | 0 (0.0) |  | 0 (0.0) |  | 3 (1.5) |  | 3 (1.5) |  | 1 (0.5) |  | 0 (0.0) |  | 0 (0.0) |  |
| Insecticide spray use in past 7 days |  | 0 (0.0) |  | 5 (2.6) |  | 5 (2.5) |  | 2 (1.0) |  | 9 (4.5) |  | 1 (0.5) |  | 0 (0.0) |  | 8 (4.0) |  |
| Survey 3, N |  | 199 |  | 199 |  | 199 |  | 198 |  | 200 |  | 193 |  | 213 |  | 194 |  |
| Malaria treatment in past 14 days^a^ |  | 3 (1.5) |  | 1 (0.5) |  | 9 (4.5) |  | 14 (7.1) |  | 14 (7.0) |  | 2 (1.0) |  | 20 (9.4) |  | 14 (7.2) |  |
| Slept under bed net night before^a^ |  | 181 (91.0) |  | 132 (66.3) |  | 122 (61.3) |  | 180 (90.9) |  | 160 (80.0) |  | 176 (91.2) |  | 178 (83.6) |  | 154 (79.4) |  |
| No use of repellent in past 7 days^b^ |  | 195 (98.0) |  | 81 (40.7) |  | 152 (76.4) |  | 193 (97.5) |  | 194 (97.0) |  | 189 (97.9) |  | 207 (97.2) |  | 181 (93.3) |  |
| Use of IRS in past 12 months^c^ |  | 0 (0.0) |  | 0 (0.0) |  | 0 (0.0) |  | 0 (0.0) |  | 0 (0.0) |  | 1 (0.5) |  | 0 (0.0) |  | 1 (0.5) |  |
| Insecticide spray use in past 7 days |  | 0 (0.0) |  | 2 (1.0) |  | 13 (6.5) |  | 2 (1.0) |  | 5 (2.5) |  | 3 (1.6) |  | 1 (0.5) |  | 2 (1.0) |  |
| Survey 4, N |  | 200 |  | 202 |  |  |  |  |  |  |  |  |  |  |  | 198 |  |
| Malaria treatment in past 14 days^a^ |  | 5 (2.5) |  | 0 (0.0) |  | - |  | - |  | - |  | - |  | - |  | 4 (2.0) |  |
| Slept under bed net night before^a^ |  | 187 (93.5) |  | 116 (57.4) |  | - |  | - |  | - |  | - |  | - |  | 152 (76.8) |  |
| No use of repellent in past 7 days^b^ |  | 199 (99.5) |  | 86 (42.6) |  | - |  | - |  | - |  | - |  | - |  | 175 (88.4) |  |
| Use of IRS in past 12 months^c^ |  | 1 (0.5) |  | 0 (0.0) |  | - |  | - |  | - |  | - |  | - |  | 1 (0.5) |  |
| Insecticide spray use in past 7 days |  | 1 (0.5) |  | 5 (2.5) |  | - |  | - |  | - |  | - |  | - |  | 5 (2.5) |  |

Y, years of age; N, number of participants included in the analyses; IRS, indoor residual spray; -, centres not included in the fourth survey.

Note: ^a^ Percentages computed without considering the missing values

^b^ No usage of mosquito coil, insecticide spray, commercial or traditional repellent.

^c^ To spray interior walls.

**Figure S1.** Prevalence of reported fever in relation to parasite density for the 5–19Y (A) and ≥20Y (B) age groups, by site

**
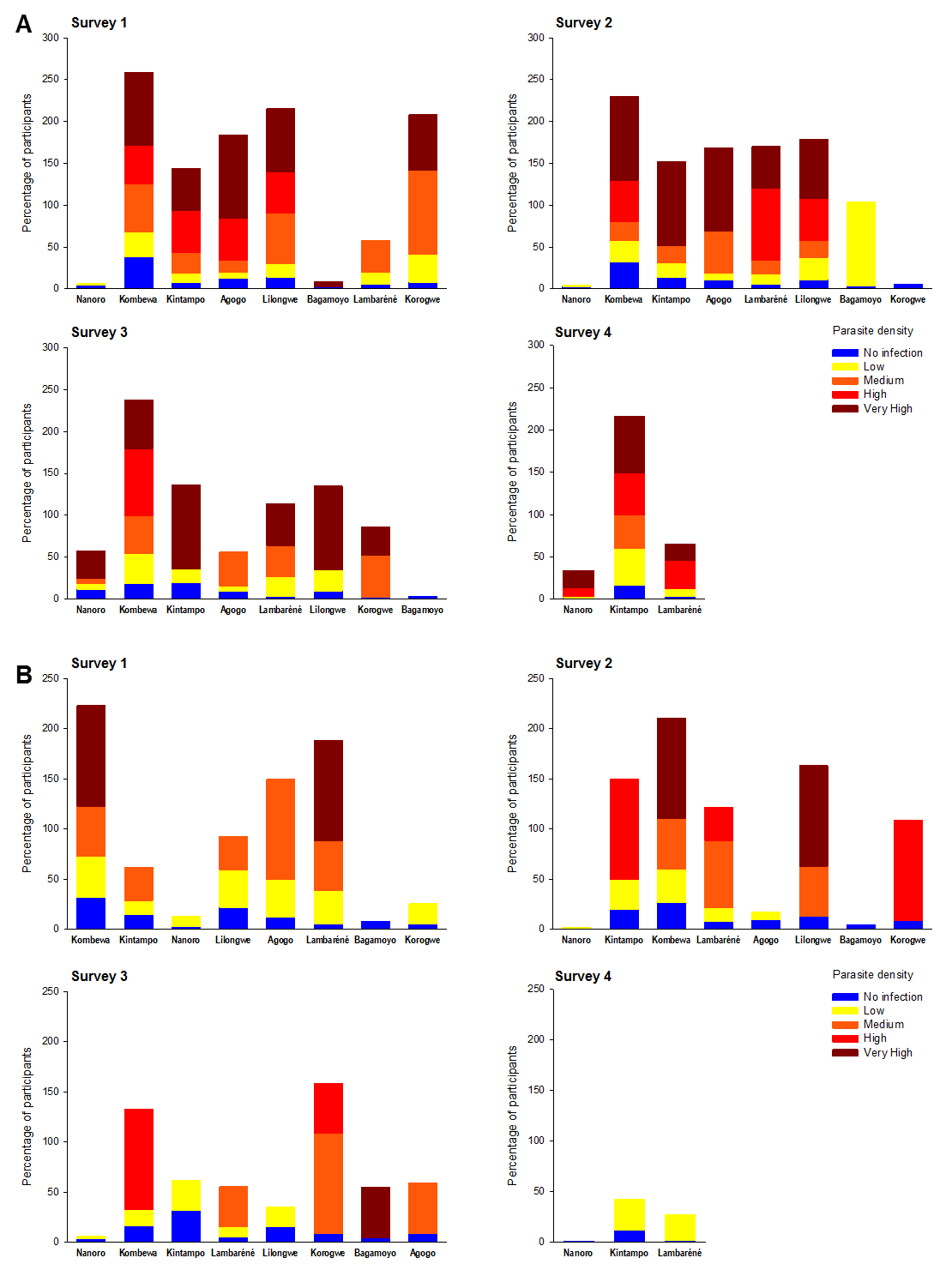
**

Y, year. Sites are ordered according to increased *Plasmodium falciparum* prevalence.

**Figure S2.** Prevalence of reported anaemia and severe anaemia, in relation to parasite density and haemoglobin concentration for the 5–19Y (A) and ≥20Y (B) age groups, by site


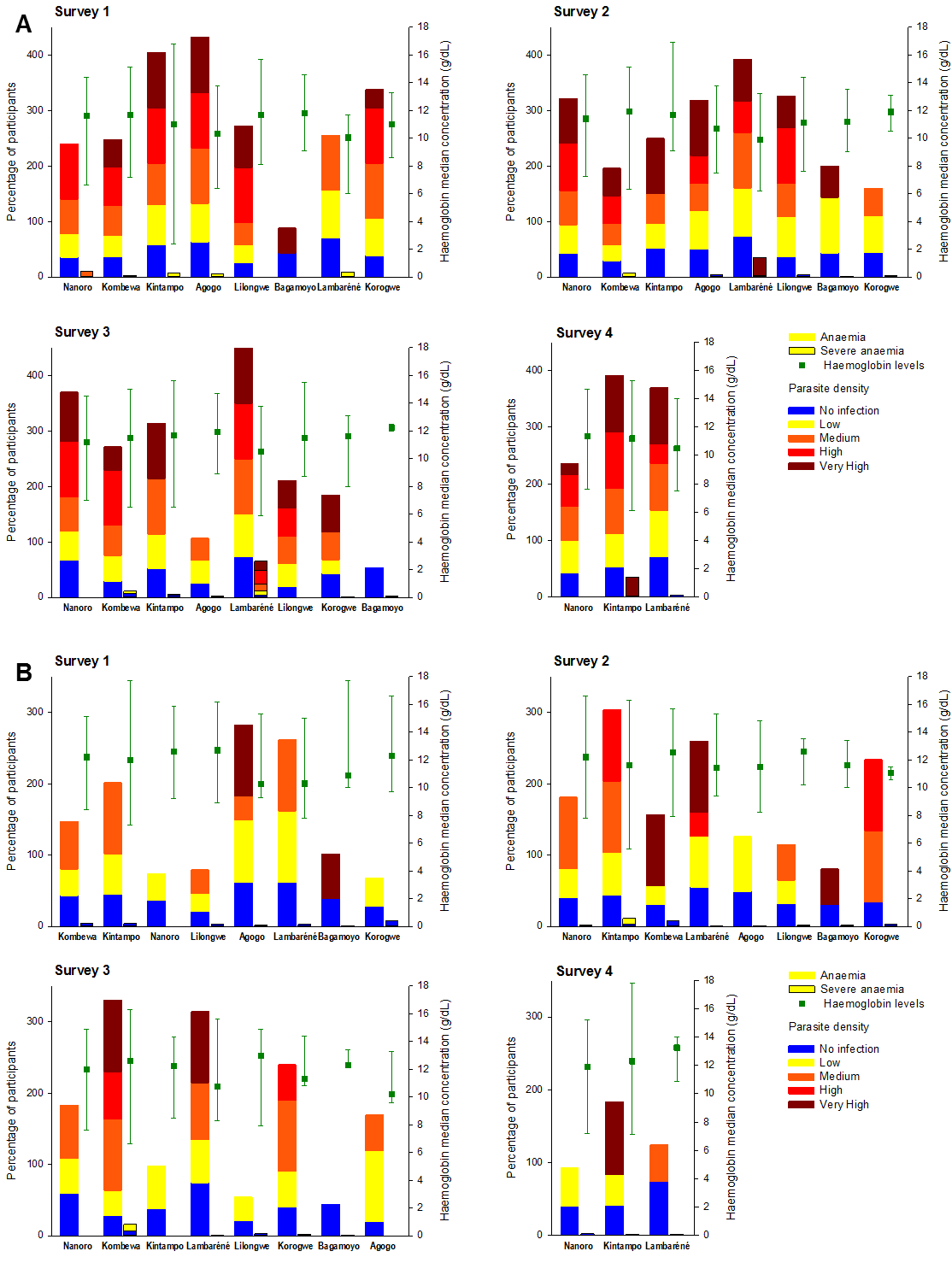


Y, year. Sites are ordered according to increased Plasmodium falciparum prevalence.
