# Supplementary material for: Longitudinal estimation of Plasmodium falciparum prevalence in relation to malaria prevention measures in six sub-Saharan African countries
Source: Malar J. 2017 Oct 27;16:433. doi: 10.1186/s12936-017-2078-3 (PMC5658967; doi:10.1186/s12936-017-2078-3)
Supplement: Supplementary file 2 — Additional file 2. Malaria rapid diagnostic test results and prevalence of infections with other parasites. [file 12936_2017_2078_MOESM2_ESM.doc]

# Additional file 2. Malaria rapid diagnostic test results and prevalence of infections with other parasites

**Table S1.** Malaria rapid diagnostic test results

|  |  | *P. falciparum* infection detected microscopically | | | | | | | |  | *P. falciparum* infection not detected microscopically | | | | | | | |
| --- | --- | --- | --- | --- | --- | --- | --- | --- | --- | --- | --- | --- | --- | --- | --- | --- | --- | --- |
|  |  | 6M–4Y | |  | 5–19Y | |  | ≥20Y | |  | 6M–4Y | |  | 5–19Y | |  | ≥20Y | |
| Site | RDT | n | % |  | n | % |  | n | % |  | n | % |  | n | % |  | n | % |
| Survey 1, N |  | 720 | |  | 446 | |  | 147 | |  | 2480 | |  | 1167 | |  | 1441 | |
| Nanoro | Positive | 213 | 95.9 |  | 105 | 98.1 |  | 22 | 75.9 |  | 18 | 9 |  | 28 | 37.8 |  | 14 | 8.4 |
|  | Negative | 9 | 4.1 |  | 2 | 1.9 |  | 7 | 24.1 |  | 183 | 91 |  | 46 | 62.2 |  | 152 | 91.6 |
| Lambaréné | Positive | 21 | 87.5 |  | 17 | 77.3 |  | 8 | 88.9 |  | 8 | 2.1 |  | 15 | 7.8 |  | 2 | 1.2 |
|  | Negative | 3 | 12.5 |  | 5 | 22.7 |  | 1 | 11.1 |  | 365 | 97.9 |  | 177 | 92.2 |  | 171 | 98.8 |
| Agogo | Positive | 87 | 94.6 |  | 38 | 95 |  | 10 | 83.3 |  | 60 | 19.7 |  | 32 | 19.6 |  | 7 | 3.7 |
|  | Negative | 5 | 5.4 |  | 2 | 5 |  | 2 | 16.7 |  | 245 | 80.3 |  | 131 | 80.4 |  | 181 | 96.3 |
| Kombewa | Positive | 168 | 96 |  | 107 | 97.3 |  | 30 | 88.2 |  | 125 | 55.6 |  | 47 | 49 |  | 29 | 17.9 |
|  | Negative | 7 | 4 |  | 3 | 2.7 |  | 4 | 11.8 |  | 100 | 44.4 |  | 49 | 51 |  | 133 | 82.1 |
| Lilongwe | Positive | 43 | 93.5 |  | 34 | 89.5 |  | 17 | 89.5 |  | 34 | 9.6 |  | 22 | 13.6 |  | 9 | 5 |
|  | Negative | 3 | 6.5 |  | 4 | 10.5 |  | 2 | 10.5 |  | 320 | 90.4 |  | 140 | 86.4 |  | 172 | 95 |
| Bagamoyo | Positive | 32 | 82.1 |  | 26 | 83.9 |  | 4 | 50 |  | 25 | 7.1 |  | 13 | 8 |  | 4 | 1.9 |
|  | Negative | 7 | 17.9 |  | 5 | 16.1 |  | 4 | 50 |  | 328 | 92.9 |  | 149 | 92 |  | 206 | 98.1 |
| Korogwe | Positive | 16 | 88.9 |  | 13 | 92.9 |  | 4 | 80 |  | 27 | 7.3 |  | 15 | 7.6 |  | 15 | 7.7 |
|  | Negative | 2 | 11.1 |  | 1 | 7.1 |  | 1 | 20 |  | 344 | 92.7 |  | 182 | 92.4 |  | 181 | 92.3 |
| Kintampo | Positive | 95 | 91.3 |  | 59 | 70.2 |  | 20 | 64.5 |  | 41 | 13.8 |  | 15 | 12.5 |  | 3 | 1.8 |
|  | Negative | 9 | 8.7 |  | 25 | 29.8 |  | 11 | 35.5 |  | 256 | 86.2 |  | 105 | 87.5 |  | 161 | 98.2 |
| Survey 2, N |  | 574 | |  | 469 | |  | 146 | |  | 2623 | |  | 1158 | |  | 1441 | |
| Nanoro | Positive | 267 | 98.2 |  | 157 | 96.9 |  | 47 | 73.4 |  | 82 | 63.1 |  | 16 | 44.4 |  | 22 | 16.2 |
|  | Negative | 5 | 1.8 |  | 5 | 3.1 |  | 17 | 26.6 |  | 48 | 36.9 |  | 20 | 55.6 |  | 114 | 83.8 |
| Lambaréné | Positive | 26 | 89.7 |  | 33 | 100 |  | 12 | 85.7 |  | 28 | 7.6 |  | 30 | 17.2 |  | 6 | 3.3 |
|  | Negative | 3 | 10.3 |  | 0 | 0 |  | 2 | 14.3 |  | 341 | 92.4 |  | 144 | 82.8 |  | 175 | 96.7 |
| Agogo | Positive | 77 | 98.7 |  | 53 | 93 |  | 12 | 92.3 |  | 47 | 14.6 |  | 35 | 24.3 |  | 15 | 8.1 |
|  | Negative | 1 | 1.3 |  | 4 | 7 |  | 1 | 7.7 |  | 275 | 85.4 |  | 109 | 75.7 |  | 171 | 91.9 |
| Kombewa | Positive | 88 | 96.7 |  | 79 | 94 |  | 15 | 83.3 |  | 69 | 22.3 |  | 32 | 27.6 |  | 22 | 12.1 |
|  | Negative | 3 | 3.3 |  | 5 | 6 |  | 3 | 16.7 |  | 240 | 77.7 |  | 84 | 72.4 |  | 160 | 87.9 |
| Lilongwe | Positive | 23 | 92 |  | 23 | 79.3 |  | 5 | 55.6 |  | 19 | 5.1 |  | 12 | 7 |  | 1 | 0.5 |
|  | Negative | 2 | 8 |  | 6 | 20.7 |  | 4 | 44.4 |  | 355 | 94.9 |  | 160 | 93 |  | 190 | 99.5 |
| Bagamoyo | Positive | 5 | 62.5 |  | 14 | 93.3 |  | 2 | 50 |  | 6 | 1.5 |  | 4 | 2 |  | 3 | 1.6 |
|  | Negative | 3 | 37.5 |  | 1 | 6.7 |  | 2 | 50 |  | 384 | 98.5 |  | 200 | 98 |  | 187 | 98.4 |
| Korogwe | Positive | 3 | 75 |  | 3 | 60 |  | 1 | 50 |  | 0 | 0 |  | 0 | 0 |  | 0 | 0 |
|  | Negative | 1 | 25 |  | 2 | 40 |  | 1 | 50 |  | 395 | 100 |  | 196 | 100 |  | 198 | 100 |
| Kintampo | Positive | 65 | 97 |  | 71 | 84.5 |  | 16 | 72.7 |  | 106 | 31.7 |  | 28 | 24.1 |  | 31 | 17.5 |
|  | Negative | 2 | 3 |  | 13 | 15.5 |  | 6 | 27.3 |  | 228 | 68.3 |  | 88 | 75.9 |  | 146 | 82.5 |
| Survey 3, N |  | 605 | |  | 483 | |  | 193 | |  | 2588 | |  | 1129 | |  | 1402 | |
| Nanoro | Positive | 261 | 99.6 |  | 162 | 98.8 |  | 75 | 88.2 |  | 113 | 85 |  | 30 | 71.4 |  | 35 | 30.7 |
|  | Negative | 1 | 0.4 |  | 2 | 1.2 |  | 10 | 11.8 |  | 20 | 15 |  | 12 | 28.6 |  | 79 | 69.3 |
| Lambaréné | Positive | 34 | 94.4 |  | 32 | 91.4 |  | 16 | 100 |  | 17 | 4.7 |  | 14 | 8.4 |  | 4 | 2.2 |
|  | Negative | 2 | 5.6 |  | 3 | 8.6 |  | 0 | 0 |  | 346 | 95.3 |  | 153 | 91.6 |  | 179 | 97.8 |
| Agogo | Positive | 52 | 86.7 |  | 35 | 87.5 |  | 3 | 100 |  | 30 | 8.8 |  | 12 | 7.4 |  | 3 | 1.5 |
|  | Negative | 8 | 13.3 |  | 5 | 12.5 |  | 0 | 0 |  | 309 | 91.2 |  | 150 | 92.6 |  | 193 | 98.5 |
| Kombewa | Positive | 107 | 97.3 |  | 103 | 94.5 |  | 31 | 86.1 |  | 120 | 41.2 |  | 40 | 43.5 |  | 26 | 16 |
|  | Negative | 3 | 2.7 |  | 6 | 5.5 |  | 5 | 13.9 |  | 171 | 58.8 |  | 52 | 56.5 |  | 136 | 84 |
| Lilongwe | Positive | 11 | 78.6 |  | 17 | 94.4 |  | 10 | 66.7 |  | 11 | 2.8 |  | 11 | 6 |  | 8 | 4.3 |
|  | Negative | 3 | 21.4 |  | 1 | 5.6 |  | 5 | 33.3 |  | 375 | 97.2 |  | 171 | 94 |  | 177 | 95.7 |
| Bagamoyo | Positive | 4 | 80 |  | 1 | 50 |  | 2 | 66.7 |  | 12 | 3.1 |  | 7 | 3.4 |  | 6 | 3.2 |
|  | Negative | 1 | 20 |  | 1 | 50 |  | 1 | 33.3 |  | 381 | 96.9 |  | 199 | 96.6 |  | 183 | 96.8 |
| Korogwe | Positive | 9 | 90 |  | 8 | 88.9 |  | 5 | 100 |  | 23 | 5.9 |  | 9 | 5.1 |  | 8 | 3.8 |
|  | Negative | 1 | 10 |  | 1 | 11.1 |  | 0 | 0 |  | 367 | 94.1 |  | 169 | 94.9 |  | 200 | 96.2 |
| Kintampo | Positive | 101 | 93.5 |  | 87 | 82.1 |  | 20 | 66.7 |  | 101 | 34.6 |  | 21 | 21 |  | 24 | 14.6 |
|  | Negative | 7 | 6.5 |  | 19 | 17.9 |  | 10 | 33.3 |  | 191 | 65.4 |  | 79 | 79 |  | 140 | 85.4 |
| Survey 4, N |  | 345 | |  | 278 | |  |  | 92 |  | 848 | |  | 328 | |  | 508 | |
| Nanoro | Positive | 212 | 99.5 |  | 141 | 99.3 |  | 49 | 96.1 |  | 147 | 79 |  | 38 | 64.4 |  | 59 | 39.6 |
|  | Negative | 1 | 0.5 |  | 1 | 0.7 |  | 2 | 3.9 |  | 39 | 21 |  | 21 | 35.6 |  | 90 | 60.4 |
| Lambaréné | Positive | 22 | 81.5 |  | 23 | 92 |  | 6 | 100 |  | 39 | 10.5 |  | 29 | 16.8 |  | 16 | 8.2 |
|  | Negative | 5 | 18.5 |  | 2 | 8 |  | 0 | 0 |  | 333 | 89.5 |  | 144 | 83.2 |  | 180 | 91.8 |
| Kintampo | Positive | 90 | 85.7 |  | 94 | 84.7 |  | 21 | 60 |  | 91 | 31.4 |  | 17 | 17.7 |  | 16 | 9.8 |
|  | Negative | 15 | 14.3 |  | 17 | 15.3 |  | 14 | 40 |  | 199 | 68.6 |  | 79 | 82.3 |  | 147 | 90.2 |

M, month; Y, year; n (%), number/percentage of participants in a given category; RDT, rapid diagnostic test; N, number of participants with available results in each category.

**Table S2.** Prevalence of *P. malaria*, *P. vivax* and *P. ovale*

|  |  | 6M–4Y | | |  | 5–19Y | | |  | ≥20Y | | |
| --- | --- | --- | --- | --- | --- | --- | --- | --- | --- | --- | --- | --- |
|  |  | % | 95% CI | |  | % | 95% CI | |  | % | 95% CI | |
|  |  |  | LL | UL |  |  | LL | UL |  |  | LL | UL |
| Survey 1, N |  | 3200 | | |  | 1613 | | |  | 1588 | | |
| Nanoro | *P. malaria* | 3.3 | 1.8 | 5.5 |  | 6.1 | 3.1 | 10.6 |  | 0 | 0 | 1.9 |
|  | *P. ovale* | 0.2 | 0 | 1.3 |  | 0 | 0 | 2 |  | 0 | 0 | 1.9 |
| Kombewa | *P. malaria* | 0.3 | 0 | 1.4 |  | 0 | 0 | 1.8 |  | 0 | 0 | 1.9 |
| Lilongwe | *P. malaria* | 0 | 0 | 0.9 |  | 2.5 | 0.8 | 5.7 |  | 0 | 0 | 1.8 |
| Bagamoyo | *P. malaria* | 0 | 0 | 0.9 |  | 0.5 | 0 | 2.8 |  | 0 | 0 | 1.7 |
|  | *P. ovale* | 0 | 0 | 0.9 |  | 0.5 | 0 | 2.8 |  | 0 | 0 | 1.7 |
| Kintampo | *P. malaria* | 1 | 0.3 | 2.5 |  | 2 | 0.5 | 4.9 |  | 0.5 | 0 | 2.8 |
|  | *P. ovale* | 0.5 | 0.1 | 1.8 |  | 0 | 0 | 1.8 |  | 0 | 0 | 1.9 |
| Survey 2, N | | 3197 | | |  | 1627 | | |  | 1587 | | |
| Nanoro | *P. malaria* | 4 | 2.3 | 6.4 |  | 7.6 | 4.3 | 12.2 |  | 0 | 0 | 1.8 |
|  | *P. ovale* | 0.2 | 0 | 1.4 |  | 0 | 0 | 1.8 |  | 0 | 0 | 1.8 |
| Lambaréné | *P. ovale* | 0.3 | 0 | 1.4 |  | 0 | 0 | 1.8 |  | 0 | 0 | 1.9 |
| Agogo | *P. ovale* | 0.5 | 0.1 | 1.8 |  | 0.5 | 0 | 2.7 |  | 0 | 0 | 1.8 |
| Kombewa | *P. malaria* | 1.8 | 0.7 | 3.6 |  | 3 | 1.1 | 6.4 |  | 0.5 | 0 | 2.8 |
|  | *P. ovale* | 0.3 | 0 | 1.4 |  | 0.5 | 0 | 2.8 |  | 1 | 0.1 | 3.6 |
| Lilongwe | *P. malaria* | 0 | 0 | 0.9 |  | 0.5 | 0 | 2.7 |  | 0.5 | 0 | 2.8 |
| Kintampo | *P. malaria* | 2.2 | 1 | 4.2 |  | 2.5 | 0.8 | 5.7 |  | 0 | 0 | 1.8 |
|  | *P. ovale* | 0.2 | 0 | 1.4 |  | 0.5 | 0 | 2.8 |  | 0 | 0 | 1.8 |
| Survey 3, N | | 3193 | | |  | 1612 | | |  | 1959 | | |
| Nanoro | *P. malaria* | 4.3 | 2.5 | 6.8 |  | 5.8 | 3 | 10 |  | 0 | 0 | 1.8 |
|  | *P. ovale* | 0.3 | 0 | 1.4 |  | 0 | 0 | 1.8 |  | 0 | 0 | 1.8 |
| Agogo | *P. malaria* | 0.3 | 0 | 1.4 |  | 0 | 0 | 1.8 |  | 0 | 0 | 1.8 |
| Kombewa | *P. malaria* | 6.2 | 4.1 | 9.1 |  | 5 | 2.4 | 9 |  | 0 | 0 | 1.8 |
|  | *P. ovale* | 0.5 | 0.1 | 1.8 |  | 2 | 0.5 | 5 |  | 0 | 0 | 1.8 |
| Kintampo | *P. malaria* | 1.3 | 0.4 | 2.9 |  | 2.4 | 0.8 | 5.6 |  | 0 | 0 | 1.9 |
|  | *P. ovale* | 0.8 | 0.2 | 2.2 |  | 0 | 0 | 1.8 |  | 0.5 | 0 | 2.8 |
| Survey 4, N | | 1193 | | |  | 606 | | |  | 600 | | |
| Nanoro | *P. malaria* | 6.5 | 4.3 | 9.4 |  | 10 | 6.2 | 14.9 |  | 0 | 0 | 1.8 |
|  | *P. ovale* | 0.3 | 0 | 1.4 |  | 0.5 | 0 | 2.7 |  | 0 | 0 | 1.8 |
| Kintampo | *P. malaria* | 1.5 | 0.6 | 3.3 |  | 3.9 | 1.7 | 7.5 |  | 0 | 0 | 1.8 |
|  | *P. ovale* | 0.5 | 0.1 | 1.8 |  | 1 | 0.1 | 3.4 |  | 0.5 | 0 | 2.8 |

M, month; Y, year; %, percentage of participants in each category; CI, confidence interval; LL, lower limit; UL, upper limit; N, number of participants included in the analyses.

Note: Sites where the prevalence for all species was 0 are not shown.
