# Supplementary material for: Longitudinal estimation of Plasmodium falciparum prevalence in relation to malaria prevention measures in six sub-Saharan African countries
Source: Malar J. 2017 Oct 27;16:433. doi: 10.1186/s12936-017-2078-3 (PMC5658967; doi:10.1186/s12936-017-2078-3)
Supplement: Supplementary file 3 — Additional file 3. Odds ratios analysis of P. falciparum infection in relation to reported usage of anti-malarial treatment and prevention. [file 12936_2017_2078_MOESM3_ESM.doc]

# Additional file 3. Associations between *P. falciparum* infection and anaemia, anti-malarial treatment and prevention measures, by site

**Table S1.** Associations between positive parasitaemia and anaemia using logistic regression

|  |  | Odds ratio (95% confidence interval); reference Yes vs. No | | | | | | | | | | | | | | |  |
| --- | --- | --- | --- | --- | --- | --- | --- | --- | --- | --- | --- | --- | --- | --- | --- | --- | --- |
|  |  | Burkina Faso, Nanoro |  | Gabon, Lambaréné |  | Ghana, Agogo |  | Kenya, Kombewa |  | Malawi, Lilongwe |  | Tanzania, Bagamoyo |  | Tanzania, Korogwe |  | Ghana, Kintampo |  |
| Survey 1 |  | 1.46 (1.11–1.94) |  | 3.49 (1.47–8.26) |  | 3.55 (2.14–5.90) |  | 1.77 (1.32–2.36) |  | 2.71 (1.78–4.12) |  | 1.35 (0.84–2.15) |  | 2.71 (1.32–5.56) |  | 2.55 (1.78–3.64) |  |
| Survey 2 |  | 1.89 (1.41–2.53) |  | 1.81 (1.01–3.26) |  | 2.60 (1.73–3.91) |  | 1.66 (1.19–2.29) |  | 2.59 (1.52–4.45) |  | 1.43 (0.66–3.11) |  | 4.08 (0.88–18.98) |  | 1.46 (1.03–2.07) |  |
| Survey 3 |  | 1.21 (0.89–1.65) |  | 2.23 (1.16–4.29) |  | 2.69 (1.76–4.10) |  | 2.05 (1.51–2.77) |  | 1.86 (1.06–3.37) |  | 0.30 (0.08–1.17) |  | 1.68 (0.73–3.89) |  | 2.37 (1.71–3.28) |  |
| Survey 4 |  | 2.38 (1.77–3.18) |  | 1.40 (0.75–2.60) |  |  |  |  |  |  |  |  |  |  |  | 1.60 (1.16–2.20) |  |

**Table S2.** Odds ratios analysis of *P. falciparum* infection in relation to reported usage of anti-malarial treatment and prevention measures, by site

|  |  | Odds ratio (95% confidence interval) | | | | | | | | | | | | | | |  |
| --- | --- | --- | --- | --- | --- | --- | --- | --- | --- | --- | --- | --- | --- | --- | --- | --- | --- |
|  |  | Burkina Faso, Nanoro |  | Gabon, Lambaréné |  | Ghana, Agogo |  | Kenya, Kombewa |  | Malawi, Lilongwe |  | Tanzania, Bagamoyo |  | Tanzania, Korogwe |  | Ghana, Kintampo |  |
| Survey 1 |  |  |  |  |  |  |  |  |  |  |  |  |  |  |  |  |  |
| No usage of malaria repellent in past 7 days |  | 3.7 (0.8–17.3) |  | 0.9 (0.5–1.5) |  | 1.4 (1.0–2.0) |  | 2.0 (0.7–5.6) |  | 0.6 (0.2–1.9) |  | - |  | 1.0 (0.2–4.4) |  | 3.5 (2.2–5.5) |  |
| Slept under bednet night before |  | 0.8 (0.6–1.1) |  | 0.6 (0.3–1.0) |  | 1.0 (0.7–1.5) |  | 0.7 (0.5–1.0) |  | 0.4 (0.3–0.7) |  | 0.8 (0.3–2.0) |  | 0.6 (0.3–1.1) |  | 1.2 (0.8–1.6) |  |
| Malaria treatment in past 14 days |  | - |  | 2.0 (0.4–8.8) |  | 0.4 (0.2–0.9) |  | 0.4 (0.3–0.7) |  | 0.6 (0.3–1.3) |  | 0.4 (0.1–2.9) |  | 0.7 (0.2–3.0) |  | 0.2 (0.1–0.5) |  |
| Use of IRS in past 12 months (to spray walls) |  | - |  | - |  | 0.9 (0.4–1.7) |  | 0.9 (0.2–3.8) |  | - |  | - |  | - |  | 0.7 (0.1–3.1) |  |
| Insecticide spray use in past 7 days |  | 0.2 (0.0–2.1) |  | - |  | 0.4 (0.2–0.9) |  | 3.1 (0.6–16.8) |  | - |  | - |  | 3.0 (0.4–25.1) |  | 0.2 (0.1–0.6) |  |
| Survey 2 |  |  |  |  |  |  |  |  |  |  |  |  |  |  |  |  |  |
| No usage of malaria repellent in past 7 days^a^ |  | - |  | 0.9 (0.6–1.5) |  | 0.8 (0.5–1.2) |  | 2.1 (0.6–7.0) |  | 1.2 (0.6–2.2) |  | - |  | - |  | 1.2 (0.8–1.9) |  |
| Slept under bednet night before^b^ |  | 1.0 (0.7–1.3) |  | 0.7 (0.5–1.2) |  | 0.9 (0.6–1.3) |  | 0.4 (0.2–0.6) |  | 0.2 (0.1–0.4) |  | 0.6 (0.2–1.9) |  | - |  | 1.2 (0.9–1.7) |  |
| Malaria treatment in past 14 days^b^ |  | 0.1 (0.0–1.0) |  | 0.7 (0.2–2.9) |  | 0.7 (0.3–1.4) |  | 0.5 (0.3–0.9) |  | 0.8 (0.2–3.2) |  | 2.7 (0.6–11.9) |  | 1.7 (0.2–13.9) |  | 0.4 (0.2–0.7) |  |
| Use of IRS in past 12 months (to spray walls) |  | - |  | - |  | 4.4 (0.3–71.2) |  | 0.2 (0.0–1.6) |  | - |  | - |  | - |  | - |  |
| Insecticide spray use in past 7 days |  | - |  | 0.5 (0.1–3.8) |  | 0.6 (0.2– 2.0) |  | 0.5 (0.1–3.7) |  | 0.4 (0.1–2.7) |  | - |  | - |  | 0.7 (0.3–1.8) |  |
| Survey 3 |  |  |  |  |  |  |  |  |  |  |  |  |  |  |  |  |  |
| No usage of malaria repellent in past 7 days^a^ |  | 1.4 (0.5–3.8) |  | 0.9 (0.5–1.3) |  | 1.6 (0.9–2.7) |  | 1.4 (0.6–3.4) |  | 0.6 (0.2–2.1) |  | - |  | 0.7 (0.1–5.4) |  | 1.6 (0.9–3.0) |  |
| Slept under bednet night before^b^ |  | 0.6 (0.4–1.0) |  | 0.7 (0.5–1.1) |  | 1.1 (0.7–1.7) |  | 0.4 (0.3–0.7) |  | 0.7 (0.4–1.3) |  | 0.5 (0.1–2.2) |  | 0.5 (0.2–1.3) |  | 0.7 (0.5–1.0) |  |
| Malaria treatment in past 14 days^b^ |  | 0.3 (0.1–0.6) |  | - |  | 1.0 (0.5–2.0) |  | 0.6 (0.4–0.9) |  | 0.7 (0.2–3.0) |  | - |  | 1.0 (0.2–4.3) |  | 0.4 (0.2–0.7) |  |
| Use of IRS in past 12 months (to spray walls) |  | - |  | - |  | - |  | - |  | - |  | - |  | - |  | 2.3 (0.3–16.3) |  |
| Insecticide spray use in past 7 days |  | - |  | 2.8 (0.6–14.0) |  | 0.3 (0.1–1.4) |  | 1.1 (0.3–3.6) |  | 1.1 (0.1–8.3) |  | - |  | - |  | 1.7 (0.4–7.7) |  |
| Survey 4 |  |  |  |  |  |  |  |  |  |  |  |  |  |  |  |  |  |
| No usage of malaria repellent in past 7 days^a^ |  | 2.8 (0.7–10.6) |  | 0.6 (0.3–1.0) |  | - |  | - |  | - |  | - |  | - |  | 1.3 (0.8–2.0) |  |
| Slept under bednet night before^b^ |  | 0.8 (0.4–1.4) |  | 0.3 (0.2–0.6) |  | - |  | - |  | - |  | - |  | - |  | 0.9 (0.6–1.3) |  |
| Malaria treatment in past 14 days^b^ |  | 0.2 (0.1–0.5) |  | - |  | - |  | - |  | - |  | - |  | - |  | 0.3 (0.2–0.7) |  |
| Use of IRS in past 12 months (to spray walls) |  | 2.0 (0.2–21.5) |  | - |  | - |  | - |  | - |  | - |  | - |  | - |  |
| Insecticide spray use in past 7 days |  | 0.2 (0.0–1.7) |  | 0.7 (0.1–5.1) |  | - |  | - |  | - |  | - |  | - |  | 0.6 (0.2–1.7) |  |

Note: ^a^ Percentages computed without considering the missing values

^b^ No usage of mosquito coil, insecticide spray, commercial or traditional repellent.

IRS, indoor residual spray; -, results not available or insufficient data for a correct estimate.
